# Fingerprinting Single and Clustered Cu Sites in Metalated MOF Catalysts via TD-DFT and In Situ Diffuse Reflectance UV–Vis Spectroscopy

**DOI:** 10.1021/acsami.5c10338

**Published:** 2025-09-08

**Authors:** Soufiane Bahou, Riya Sehrawat, Bunyarat Rungtaweevoranit, Ali M. Abdel-Mageed, Ashour A. Ahmed

**Affiliations:** † 9187 Leibniz-Institut für Katalyse e.V. (LIKAT), Albert-Einstein-Str. 29a, Rostock 18059, Germany; ‡ National Nanotechnology Center (NANOTEC), 371383 National Science and Technology Development Agency (NSTDA), Khlong Luang, Pathum Thani 12120, Thailand; § University of Rostock, Institute of Physics, Albert-Einstein-Str. 23-24, Rostock 18059, Germany

**Keywords:** single-atom catalysts, Cu active sites, metal nuclearity, Zr-node defects, in situ spectroscopy, time-dependent DFT (TD-DFT), theory−experiment correlation, electronic structure fingerprinting

## Abstract

Metal–organic frameworks (MOFs) are transformative platforms for heterogeneous catalysis, but distinguishing atomically dispersed metal sites from subnanometric clusters remains a major challenge. This often demands the integration of multiple characterization techniques, many of which either lack the resolving power to distinguish active sites from their surrounding environments (e.g., low Z-contrast for light elements in electron microscopy) or are costly and require tedious data processing (e.g., X-ray absorption spectroscopy). Here, we introduce an integrated diffuse reflectance UV–vis spectroscopy and time-dependent DFT approach to overcome these limitations, enabling in situ discrimination of Cu centers installed into Zr-based UiO-66. By systematically modeling isolated Cu_1_ sites and clustered Cu_*x*_ species (*x* = 2–8), we decode optical fingerprints tied to nuclearity, oxidation states (Cu^0^, Cu­(I), Cu­(II)), and ligand coordination shells. Quantitative analysis reveals that the Cu_1_/UiO-66 catalyst maintains a single-site-dominated landscape (Cu­(I): 52–77%), with minor Cu_2_ (12–18%) and Cu_3_ (12–27%) contributions. In contrast, the Cu_*x*_/UiO-66 catalyst exhibits a dynamic multisite environment, balancing Cu­(I) (46–63%) with Cu_3_ clusters (36–48%) and variable Cu_2_ (0–8%). This approach resolves the spatial and temporal gaps of conventional and high-resolution techniques, offering a cost-effective and atomically precise strategy to correlate spectral signatures with active site architectures. This contribution establishes a broadly applicable pathway to characterize and modulate MOF-based catalysts with tunable optical and catalytic properties for sustainable energy, chemical transformations, and optoelectronics.

## Graphical abstract

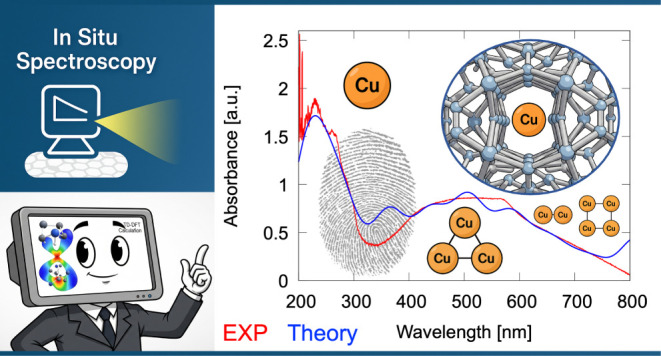

## 1 Introduction

The sustainable development of modern society mandates a sustainable supply of renewable energy to gradually replace dwindling fossil resources and thus limit greenhouse gas emissions.^1^ In this context, porous materials have become indispensable in industrial processes such as catalysis, selective separation, and petrochemistry.^2^ Among these materials, such as zeolites, clays, carbon materials, and high-surface-area oxides, metal–organic frameworks (MOFs) have emerged over the past two decades as promising candidates for many applications, including catalysis,^3^ gas adsorption,^4^ and ambient water capture.^5,6^ Their exceptional surface area, tunable functionality, and atomic-level structural precision make them ideal platforms for designing advanced heterogeneous catalysts,^7^ especially when considering their regular crystalline structure.^4,8^ This feature significantly enhances the ability to elucidate the structure of surface active sites (surface ensembles), widely recognized as one of the primary challenges in heterogeneous catalysis.^9^

Although concerns about thermal stability initially limited their catalytic applications, several MOFs have demonstrated so far excellent stability and performance in key reactions such as CO oxidation,^10,11^ partial methane oxidation,^12^ and CO_2_ hydrogenation.^13^ Among these, UiO-66, a Zr-based MOF, has attracted considerable attention. Its framework consists of zirconium oxide nodes linked by terephthalic acid (1,4-benzenedicarboxylic acid, BDC) ligands, forming a face-centered cubic lattice with an *Fm–3m* space group and a lattice parameter of 20.7 Å.^14^ The structure features a 7.5 Å tetrahedral cage, a 12 Å octahedral cage, and a 6 Å pore aperture. Each zirconium oxide node exhibits a cuboctahedral geometry, coordinating up to 12 BDC ligands.

One of the distinct features of the UiO-66 framework is its tendency to form molecular defects, which often serve as crucial sites for binding active metal centers.^15^ This framework is characterized by two types of defects: missing-ligand defects and missing-cluster defects. Missing-ligand defects can generate additional zirconium metal sites terminated with labile ligands, which are beneficial for catalytic reactions, while missing-cluster defects enhance the framework’s accessibility by increasing pore size and surface area.^16^ Leveraging these intrinsic defect sites, recent studies have demonstrated that atomically dispersed copper species can be incorporated into UiO-66, significantly enhancing its performance in CO oxidation and other catalytic processes.^11,17,18^

Reducing metal nanoparticles in supported catalysts to the atomic scale has long been a central goal in heterogeneous catalysis due to advantages such as increasing the fraction of active sites and the possibility of better controlling product selectivity for targeted reactions. However, stabilizing small clusters or individual metal atoms and preventing their migration and sintering under catalytic reaction conditions remains a major challenge, often leading to catalyst deactivation, particularly at elevated temperatures. Taking advantage of the intrinsic defect sites in UiO-66, Abdel-Mageed et al. recently demonstrated that isolated Cu ions can be incorporated into the Zr-based UiO-66 framework, achieving high performance in CO oxidation and preferential CO oxidation.^11^ However, a reliable confirmation of Cu as single sites demands the use of a multiple of complementary techniques, applied together with extensive data analysis. This challenge is common to the majority of single-atom catalysts.

In this study, we aim to resolve and differentiate between copper single atoms (Cu_1_) and clustered species (Cu_*x*_) embedded in the UiO-66 framework. Conventional spectroscopic techniques often struggle to resolve these species due to their subtle structural and electronic differences. For instance, aberration-corrected TEM is powerful for imaging cluster size but offers limited sensitivity for elements with similar Z-contrast, while X-ray absorption spectroscopy (XAS/EXAFS) provides valuable information on oxidation state and local geometry but typically requires synchrotron access, is costly to perform, and demands complex data processing. Likewise, vibrational spectroscopies such as FT-IR and Raman spectroscopy are invaluable for probing ligand binding modes, framework phonons, and local bonding environments. This also requires the application of probe molecules such as CO and NO and very careful corrections of matrix background dependent on reaction conditions to gain comparatively less unambiguous information into the subtle electronic-structure rearrangements that distinguish different metal nuclearity or oxidation states, and therefore benefits from integration with complementary, electronically sensitive techniques.^19^

Advantageously, DR–UV–vis spectroscopy, particularly when integrated with time-dependent density functional theory (TD-DFT) modeling and analysis, provides a versatile, accessible, and in situ-capable approach with high temporal resolution, without requiring large-scale facilities.^20,21^ Importantly, its implementation can be readily achieved on widely available UV–vis spectrometers when coupled with diffuse reflectance cells. This combined approach not only discriminates Cu single sites (e.g., Cu­(I) vs Cu­(II)) from small Cu clusters with differing oxidation states, spin multiplicities, and nuclearities anchored at the Zr nodes of UiO-66, but also monitors their electronic and structural evolution under reaction-relevant conditions. By probing low-energy electronic excitations, including d–d transitions and charge-transfer processes such as metal-to-ligand charge transfer (MLCT), ligand-to-metal charge transfer (LMCT), and metal-to-metal charge transfer (MMCT), which are exquisitely sensitive to oxidation state, Cu–Cu coupling, ligand π-conjugation, and local coordination geometry (e.g., bridges, terminal hydroxyls, unsaturated Zr–O–Cu linkages), DR–UV–vis reveals how subtle changes in coordination environment and nuclearity govern active-site reactivity and interfacial electron-transfer pathways.^21^ This capability enables robust spectral fingerprinting and, critically, the possible correlation of site-specific electronic descriptors with catalytic turnover, selectivity, and stability under realistic reaction conditions that are easily accessible with operando DR–UV–vis.

Our modeling approach systematically explores the influence of computational factors (e.g., DFT functionals and basis sets) and physical descriptors (e.g., nuclearity, oxidation state, spin multiplicity, and ligand environment) on the spectroscopic behavior of Cu-based sites. Direct comparison between calculated and in situ DR–UV–vis spectra uncovers critical insights into the evolution, coordination, and electronic structures of Cu active sites under working conditions. This methodology enables real-time discrimination between single-atom and clustered species and provides a robust basis for the rational design of advanced copper-based MOF catalysts.

In the following sections, we detail the synthesis and characterization of Cu/UiO-66 samples, outline the TD-DFT modeling strategy, present experimental and simulated spectral correlations, and discuss the implications for active-site identification.

## 2 Methods

### 2.1 Experimental Methods

#### 2.1.1 Catalyst Preparation of UiO-66, Cu_*x*_/UiO-66, and Cu_1_/UiO-66

All chemicals used in the preparation of Cu/UiO-66 catalysts were purchased from Sigma-Aldrich. For the synthesis of UiO-66, we followed identical procedures reported in earlier publications.^10,11^ Briefly, we mixed 1,4-benzenedicarboxylic acid (25 mg, 98%), zirconium tetrachloride (33.4 mg, 99.5%), a solution of DMF (10 mL), and acetic acid (0.7 mL) in a 20 mL scintillation vial, which was sealed and heated at 120 °C for 1 day. The reaction product was then centrifuged (10,000 rpm), washed with DMF three times followed by acetone three times and dried under dynamic vacuum overnight (see the synthesis scheme illustrated in Figure S1).

For the Cu_*x*_/UiO-66 catalyst, we dissolved 3.3 g of Cu­(NO_3_)_2_·3H_2_O in DMF (225 mL) in a 500 mL media bottle. Triethylamine (1.25 mL) was added dropwise to the solution while stirring rapidly (200 rpm). UiO-66 powder was subsequently added to the solution, and the solution was briefly sonicated to disperse the MOF powder. Then, the suspension was stirred overnight at room temperature. Cu_*x*_/UiO-66 was collected using a centrifuge (10,000 rpm, 5 min) and washed with DMF (45 mL) over a 48-h period and with acetone (45 mL) over another 24-h period. UiO-66-Cu was then dried under dynamic vacuum overnight at room temperature.

For the Cu_1_/UiO-66 catalyst, 540 mg of Cu­(Cl)_2_·2H_2_O was dissolved in 9 mL of DMF in a 20 mL scintillation vial. Afterward, 600 mg of UiO-66 was dispersed into the Cu^2+^ solution. To ensure a proper seal, PTFE tape was applied to the vial’s threads. The vial was then sealed, placed in a preheated isothermal oven at 85 °C, and held at that temperature for 24 h. Afterward, the product was recovered by centrifugation at 10,000 rpm, followed by four sequential washes with 30  mL of DMF over a 24-h period. This was followed by four washes with 30 mL of acetone. Finally, the catalyst was dried under dynamic vacuum and subsequently dried in the oven at room temperature (see Figure S2).

#### 2.1.2 Structural Characterization

##### 2.1.2.1 Inductively Coupled Plasma Optical Emission Spectroscopy (ICP-OES)

ICP-OES was used to determine the copper loadings on Cu_*x*_/UiO-66 and Cu_1_/UiO-66 using a Varian 715-ES ICP-OES (Varian, Palo Alto, CA, USA). For characterization, each catalyst sample was dissolved in a mixture of hydrogen fluoride and aqua regia. The resulting solutions underwent microwave-assisted digestion at 200 °C and 60 bar.

##### 2.1.2.2 Surface Area and Porosity Measurements

The porosity of the prepared catalysts was obtained using a N_2_ adsorption experiments. Based on the generated adsorption isotherms, BET (Brunauer–Emmett–Teller) and BJH (Barrett–Joyner–Halenda) models were applied to calculate specific surface area, pore volume, and pore sizes. For the process, a Micromeritics ASAP 2010 (Accelerated Surface Area and Porosimetry System) was used to measure N_2_ adsorption isotherms at −196 °C. Prior to all measurements, the catalysts were dried and degassed at a temperature of 200 °C for 4 h to desorb any residual solvent molecules trapped in the MOF micropores.

##### 2.1.2.3 Powder X-ray Diffraction (PXRD)

PXRD measurements of the Cu_*x*_/UiO-66 and Cu_1_/UiO-66 were determined using an X’Pert3 diffractometer (Panalytical, Almelo, The Netherlands) equipped with an X’celerator semiconductor detector and a sample changer employing various apertures and monochromators. In the X-ray tube, the copper Kα1/α2 radiations were used to generate X-rays with an electron acceleration voltage of 40 kV and a current of 40 mA. The fresh and spent catalysts were attached to silicon zero-background holders, which are engineered with silicon to have low X-ray absorption and a zero-background feature to avoid producing additional peaks during the measurement process. The X-ray diffraction patterns were recorded at a fine angular interval of 0.0167° per step with a measurement time of 25 s per step to ensure accurate results. The locations and shapes of the peaks in the data were analyzed using a mathematical model called the pseudo-Voigt function, which is a combination of Gaussian and Lorentzian functions to describe the peaks for identifying the structure of the material. This analysis was performed using the HighScore Plus software package (Panalytical, Almelo, The Netherlands). In addition, the measured data were compared with the reference patterns in the PDF-2 database provided by the International Center for Diffraction Data (ICDD) to identify the phases present in the samples.

##### 2.1.2.4 In Situ Diffuse Reflectance UV–Vis (DR–UV–Vis) Spectroscopy

In situ DR–UV–vis spectra were recorded using a UV-2600 Series spectrophotometer produced by Shimadzu. The spectrophotometer is equipped with a single monochromator and a D2 (deuterium) lamp for the ultraviolet spectrum (from 185 to 370 nm) as well as a WI (halogen) lamp for the visible/near-infrared spectrum (from 370 to 900 nm or 1400 nm if equipped with optional accessories). These two types of lamps are used because they emit a smooth and continuous spectrum. The monochromator allows a band of wavelengths to pass through rather than a single wavelength. The spectrophotometer enables the measurement of absorbance for solid samples using the Praying Mantis High-Temperature Reaction Chamber, which is particularly effective for powders with large surface areas, such as UiO-66. UiO-66 can produce accurate spectrophotometric data without any dilution. After making a baseline correction, Cu_*x*_/UiO-66 and Cu_1_/UiO-66 samples were placed in the Praying Mantis chamber, and the UV–vis reflectance spectra were recorded using the LabSolutions UV–vis software. Argon gas was used to purge the sample compartment of residuals at a flow rate of 30 mL/min for all measurements. H_2_ was calibrated to 10% H_2_/Ar with a flow rate of 3 mL/min.

### 2.2 Molecular Modeling and Computational Details

#### 2.2.1 Molecular Modeling

Quantum chemical calculations were carried out to elucidate the molecular structures, as well as the electronic and spectroscopic properties, of two catalyst systems: one containing isolated Cu single atoms (Cu_1_) and the other composed of Cu subnanometric clusters (Cu_*x*_), both anchored at the Zr nodes of UiO-66 (see Figure 1). To this end, two primary classes of molecular models were developed: Cu_1_-MOF, representing mononuclear Cu sites, and Cu_3_-MOF, featuring trimeric Cu clusters. These models were designed to mimic the experimentally observed single-site and clustered Cu species in UiO-66-supported catalysts (Cu_1_/UiO-66 and Cu_*x*_/UiO-66, respectively), capturing key aspects of the coordination environment, oxidation states, and geometric topology of the active sites under varying redox conditions.^10,18,22^

**Figure 1:**
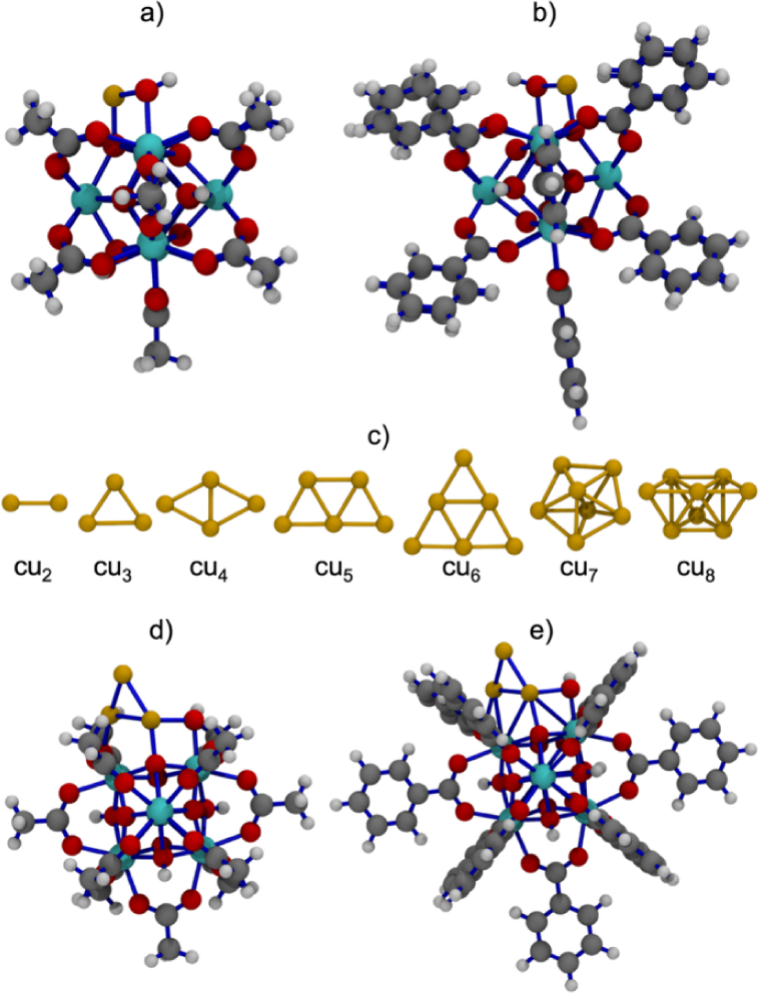
Optimized geometries of the Cu_1_-MOF modeled structures incorporating acetate (a) and benzoate (b) linkers, bare copper clusters Cu_*x*_, *x* = 2–8 (c), and Cu_3_-MOF catalysts with acetate (d) and benzoate (e) linkers. Atom colors: hydrogen (white), carbon (silver), oxygen (red), copper (golden brown), and zirconium (cyan). Bonds are highlighted in blue to aid visual distinction.

All models were constructed using a single Zr oxide (Zr_6_O_8_) node, the fundamental secondary building unit of the UiO-66 framework, where the 12 coordination sites were terminated by acetate ligands, serving as computationally efficient surrogates for the native benzene-1,4-dicarboxylate (BDC) linkers. This widely accepted simplification in MOF cluster modeling retains the essential local geometry and electronic structure while substantially reducing the computational demand. In selected cases, benzoate ligands were used to assess the effect of π-conjugation on the electronic structure and spectral features. In all models, Zr atoms were treated as Zr^4+^ ions, consistent with experimental XPS and NAP-XPS analyses.

##### 2.2.1.1 Cu_1_-MOF Models

The Cu_1_-MOF model (Figure 1a) consists of one Cu atom coordinated to the oxygen atoms of the Zr_6_O_8_ node located at the defect sites, with a molecular stoichiometry of C_22_H_37_CuO_31_Zr_6_ (97 atoms). Based on experimental insights, the copper center was primarily modeled as Cu­(I) in a singlet spin state, resulting in an overall neutral molecular system. To evaluate the influence of the oxidation state, an alternative model was built where the Cu center was treated as Cu­(II), resulting in a molecular system with a total charge of +1 and a doublet spin state. Apart from the electronic configuration, both Cu­(I) and Cu­(II) models share the same geometry and atomistic composition. Additionally, to probe the effect of linker conjugation, an extended model incorporating benzoate ligands in place of acetates was constructed (Figure 1b), yielding a larger structure with the stoichiometry C_77_H_59_CuO_31_Zr_6_ (174 atoms).

##### 2.2.1.2 Bare Cu_x_Cluster and Cu_3_-MOF Models

To investigate the influence of nuclearity and cluster size on the electronic transitions of Cu clusters supported within the UiO-66 framework (Cu_*x*_/UiO-66), we initially modeled a series of bare copper clusters (Cu_*x*_, *x* = 2–8) in isolation, without inclusion of the Zr_6_O_8_ node (see Figure 1c). These standalone cluster calculations served as a reference to understand the intrinsic electronic characteristics of Cu clusters upon increasing size. The charge and spin multiplicity of each cluster were assigned based on parity: even-numbered clusters were modeled with a spin multiplicity of 2, and odd-numbered clusters with a multiplicity of 1, while maintaining overall charge neutrality (total charge = 0) in all cases. Based on insights gained from this series, particularly regarding the spectroscopic and electronic behavior of Cu_3_, a trimeric Cu cluster was selected for incorporation into the UiO-66 framework, leading to the construction of the Cu_3_-MOF model for further investigation.

Each Cu_3_-MOF model features three Cu atoms arranged in a triangular geometry, reflecting a compact and electronically cooperative configuration (see Figure 1d–e). In these models, two Cu atoms are typically bridged by hydroxyl or carboxylate ligands and are coordinated to two Zr ions within the Zr_6_O_8_ node, preserving the structural integrity of the UiO-66 framework. The third Cu atom is directly bonded to the other two, completing the triangular Cu_3_ core through Cu–Cu interactions.

To systematically investigate the impact of the oxidation state and electronic configuration, a series of Cu_3_-MOF models were developed. These configurations represent chemically plausible species, either stable or transient, that may form under thermal treatment or reducing reaction conditions commonly encountered during catalytic operation. The specific models examined include:

Neutral Cu(0) trimer (3Cu^0^ M2): all copper atoms are treated as neutral Cu(0); total charge = 0, spin multiplicity = 2; stoichiometry: C_22_H_38_Cu_3_O_31_Zr_6_ (100 atoms).
Neutral Cu(0) trimer (3Cu^0^ M4): same as above but with spin multiplicity = 4.
Fully oxidized Cu­(I) cluster (3Cu­(I)): obtained by deprotonation of the above models (3 protons removed); total charge = 0, spin multiplicity = 1; stoichiometry: C_22_H_35_Cu_3_O_31_Zr_6_ (97 atoms).
Mixed-valence cluster (2Cu^0^ + 1Cu­(I)): total charge = 0, spin multiplicity = 1; stoichiometry: C_22_H_37_Cu_3_O_31_Zr_6_ (99 atoms).
Mixed-valence cluster (2Cu­(I) + 1Cu^0^): total charge = 0, spin multiplicity = 2; stoichiometry: C_22_H_36_Cu_3_O_31_Zr_6_ (98 atoms).

It is important to note that the reported total charges and multiplicities refer to the entire molecular system, including the Cu_3_ cluster, the Zr_6_O_8_ node, and all coordinating ligands, rather than the Cu_3_ moiety in isolation. This distinction is critical for accurately describing the electronic structure and excitation behavior of MOF-supported copper clusters in a realistic catalytic context.

In selected Cu_3_-MOF models, benzoate (Figure 1e) ligands were used instead of acetates (Figure 1d), producing significantly larger models (174–177 atoms) to assess the role of π-conjugated linkers.

#### 2.2.2 Computational Details

All geometries of the Cu_1_-MOF and Cu_3_-MOF acetate-based models were optimized using the M06L^23^ functional and Def2TZVP basis set,^24^ along with Grimme’s D3 dispersion correction^25^ using the Gaussian16 software package.^26^ Harmonic vibrational frequency analyses verified that each optimized structure corresponds to a true local minimum, as indicated by the absence of imaginary frequencies and zero gradient norms.

For larger molecular systems, involving Cu_1_-MOF and Cu_3_-MOF benzoate-based models, geometry optimizations were performed using the CP2K program package, employing the BFGS algorithm and the hybrid Gaussian and plane-wave (GPW) approach, which offers improved efficiency and scalability for extended coordination networks.^27–30^ These DFT calculations combined the PBE exchange-correlation functional^31^ with GTH pseudopotentials.^32^ An electronic density cutoff of 500 Ry was used to describe core electrons, and the double-ζ valence polarized (DZVP) basis set,^33^ optimized for use with GTH pseudopotentials, was applied. Grimme’s D3 dispersion correction was also included in these calculations.^25^

For all optimized geometries, UV–vis absorption spectra were computed by using linear-response time-dependent density functional theory (TD-DFT). The CAM-B3LYP functional,^34^ a long-range-corrected hybrid exchange-correlation functional, was predominantly employed due to its reliable performance in describing charge-transfer (CT) and d–d transitions in transition-metal complexes. Each spectrum comprised 20 singlet excited states to ensure adequate resolution of the main electronic transitions.

To evaluate the sensitivity of excitation energies and spectral profiles to computational parameters, systematic benchmarking of both DFT functionals and basis sets was conducted. Three basis sets from the Ahlrichs Def2 family—Def2SVPP, Def2TZVP, and Def2TZVPP^24,35^ —were selected to represent increasing levels of valence flexibility and polarization quality. These basis sets range from a modest split-valence double-ζ set with polarization functions on all atoms (Def2SVPP), to a triple-ζ set with polarization only on heavy atoms (Def2TZVP), and finally to a high-accuracy triple-ζ set with double polarization functions on both hydrogen and non-hydrogen atoms (Def2TZVPP). Notably, the Def2 basis sets are well-balanced, systematically constructed across the periodic table, and optimized for modern DFT methods, offering excellent accuracy for both ground- and excited-state properties. In parallel, the influence of the exchange-correlation functional was examined by comparing TD-DFT results obtained with M06L,^23^ B3LYP,^36^ CAM-B3LYP,^34^ LC-ωHPBE,^37^ and ωB97XD.^38^ All calculations were performed using the Gaussian 16 software package.^26^

## 3 Results and Discussion

### 3.1 Structural Analyses

The structural integrity of the Cu_1_/UiO-66 and Cu_*x*_/UiO-66 catalysts was examined using ICP-OES, PXRD, and N_2_ adsorption experiments, following previous protocols.^10,22^ ICP-OES (Table 1) shows that Cu_*x*_/UiO-66 contains a higher copper loading (3.95 wt %) with a Cu/Zr_6_ ratio of 1.46, consistent with a greater degree of Cu incorporation into the framework and, on average, more than one Cu atom per Zr_6_ node. By contrast, Cu_1_/UiO-66 exhibits a lower Cu loading (1.55 wt %) and a Cu/Zr_6_ ratio of 0.57. This reduced ratio suggests a more dilute copper distribution, with defect sites predominantly occupied by individual Cu atoms rather than clusters, in agreement with the intended single-site character of Cu_1_/UiO-66.

Based on the N_2_ adsorption isotherms (Table 1 and Figure 2a), we obtained a BET specific surface area (SSA_BET_) of 997 m^2^/g for Cu_*x*_/UiO-66, which is notably lower than that of the pristine UiO-66 (1325 m^2^/g) framework. This indicates that the copper loading affects the overall surface area, as the copper clusters bind to the defects, partially blocking pores and limiting N_2_ accessibility. For Cu_1_/UiO-66, the SSA_BET_ is 1146 m^2^/g, which is higher than that of Cu_*x*_/UiO-66 but still lower than that of the UiO-66 framework. These results are consistent with earlier findings showing that the incorporation of single copper atoms into UiO-66 only slightly decreases the surface area compared to copper clusters.^22^ This is attributed to minimal structural disturbance and effective defect stabilization. The increase in surface area suggests that copper is more finely dispersed and most likely distributed as isolated individual atoms rather than clusters, causing minimal obstruction to the porous network, in agreement with earlier findings. This interpretation is supported by the pore volume data, where Cu_1_/UiO-66 has about 20% larger total pore volume (0.457 cm^3^/g) than Cu_*x*_/UiO-66 (0.381 cm^3^/g), which would lead to blockage of the micropores and is thus expected to affect the specific surface area and porosity.

Careful analysis of N_2_ adsorption isotherm, employing t-plot methods to quantify microporosity (<2 nm) and BJH desorption to assess mesoporosity (2–100 nm), may provide supportive insights into the pore architecture and surface characteristics. These analyses refer to structural and functional divergences driven by differences in copper dispersion modes. Cu_1_/UiO-66 demonstrates a microporous-dominant framework with a BET surface area of 1146 m^2^/g, lower than that of pristine UiO-66 (1325 m^2^/g), yet higher than that of Cu_*x*_/UiO-66 (997 m^2^/g), even when considering a 10% experimental error in the N_2_ adsorption experiment. The t-plot analysis confirms 99% micropore contribution (1137 m^2^/g), supported by sharp incremental pore volume peaks at 0.6, 0.8, 1.1, and 1.8 nm, corresponding to tetrahedral and octahedral cavities as well as defect-induced micropores. A cumulative micropore volume of 0.456 cm^3^/g and negligible mesoporosity (0.032 cm^3^/g) reflect preserved crystallinity attributable to atomic Cu dispersion at defect sites without pore occlusion.

Conversely, Cu_*x*_/UiO-66 catalyst exhibits hierarchical porosity, balancing a reduced micropore volume (0.382 cm^3^/g) with a 4.5× higher mesopore volume (0.143 cm^3^/g). Broadened incremental pore volume peaks below 1.6 nm and a pronounced mesoporous tail (2–5 nm) signify structural distortion from Cu cluster formation, which occludes micropores and elevates external surface area (49.5 m^2^/g vs 8.9 m^2^/g for Cu_1_/UiO-66).

Adsorption behavior offers additional insights, particularly through the BET C constant, which, based on previous reports,^39–41^ provides insights into the trend variation strength of interactions between the adsorbate and the surface. Specifically, it can reflect the relative binding energy of the first adsorbed monolayer compared to subsequent layers, with higher values indicating a stronger affinity. Cu_1_/UiO-66 exhibits a notably high C value (4104), pointing to stronger interactions with N_2_ and enhanced surface affinity. In contrast, Cu_*x*_/UiO-66 shows a moderate C value (998), suggesting weaker adsorbate–surface interactions.

The XRD results (Figure 2b) of Cu_1_/UiO-66 show sharp and well-defined peaks, which are in line with the high crystallinity of this material. These peaks indicate that the structure remains intact after loading copper. There are no additional peaks associated with metallic copper or copper oxide phases, which indicates that the copper is likely present as single atoms and integrated into the UiO-66 framework by occupying the defects without forming clusters. For Cu_*x*_/UiO-66, the XRD results also show similar peaks, confirming that the UiO-66 structure remains intact. However, these peaks are more intense, indicating a higher degree of crystallinity. No characteristic reflections for Cu_*x*_ or CuO_*x*_ species are observed, suggesting that Cu is present in a highly dispersed state, well below the XRD detection limit for metal or oxide clusters (typically <2 nm). This observation aligns closely with our previous findings on similar materials.^10,22^

Additionally, the PXRD pattern of Cu_*x*_/UiO-66 shows a higher intensity in the 2θ positions compared to Cu_1_/UiO-66, which may corresponds to contributions from copper species or slight changes in crystallinity upon introduction of larger amount of Cu into the UiO-66 framework.

**Table 1:** Summary of Cu Loading, Cu/Zr_6_, Surface Areas, and Pore Volume of the Catalysts

**Catalyst**	**Cu loading (wt %)**	**Cu/Zr**_**6**_	**BET surface area (m** ^**2**^ **/g)**	**Pore volume (cm** ^**3**^ **/g)**	**Mesopore volume (BJH) (cm** ^**3**^ **/g)**	**Dominant pore size (nm)**	**C constant**
Cu_*x*_/UiO-66	3.95	1.46	997	0.382	0.143	0.6–5	4104
Cu_1_/UiO-66	1.55	0.57	1146	0.456	0.032	0.6–1.8	998

**Figure 2:**
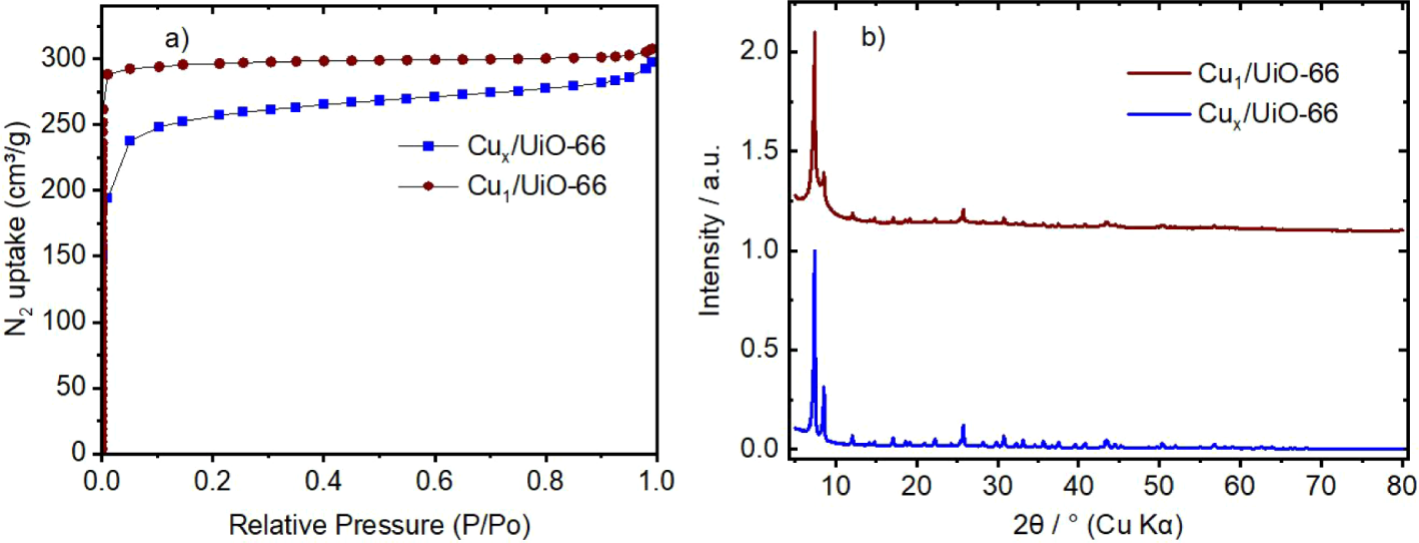
N_2_ adsorption isotherms (a) and powder X-ray diffraction patterns (b) of the fresh Cu_1_/UiO-66 and Cu_*x*_/UiO-66 catalysts.

### 3.2 In Situ UV–Vis Spectroscopy

For Cu_*x*_/UiO-66 (Figure 3a), before H_2_ reduction, the absorption peaks recorded at 230, 260, 284, and 297 nm are attributed primarily to charge transfer transitions between O^2–^ ligands and Cu^2+^ species, consistent with oxidized Cu states. These spectral features are in agreement with reported results on similar catalysts.^11,42^ In earlier results, three maxima (239, 267, and 297 nm) were identified and attributed to Cu^1+^-like states. In another study, 4 maxima (230, 263, 283, and 298 nm) were detected in the spectra, exhibiting similarities to the previous findings with a slight blue shift in the wavelengths.^22^ Such variations arise not only from differences in catalyst preparation and pretreatment but also from the local coordination environment. In UiO-66, missing-linker defects at the Zr_6_O_8_ nodes generate under-coordinated sites terminated by OH and H_2_O ligands, which perturb the ligand field symmetry and electronic surroundings of Cu species, producing measurable shifts in UV–vis bands. There are no signals above 300 nm, which indicates that no metallic Cu clusters (nanoparticles) are formed and that only Cu is in oxidized states. After reduction, a new absorption band is observed around the 567 nm region, indicating the plasmon resonance of metallic Cu clusters. This is strong evidence that significant amounts of Cu^2+^ species have been reduced to metallic Cu clusters. Notably, the 230–297 nm bands persist after reduction, indicating that some oxidized Cu remains, likely stabilized at defect sites. These results demonstrate that Zr-node defects not only anchor Cu species but also modulate their redox behavior and spectroscopic signatures.

For Cu_1_/UiO-66 (Figure 3b), the same peaks are observed at 230, 260, 284, and 297 nm before reduction, which correspond to charge transfer between O^2–^ and Cu^2+^. Compared to Cu_*x*_/UiO-66, the peaks are barrower and more sharply defined, which demonstrates the highly dispersed and isolated nature of Cu sites. In addition, the broader shoulders are not shown in the spectra, reflecting that Cu exists as single atoms rather than clusters. Furthermore, an absorption band appears at 387 nm, which can correspond to oxo dimeric Cu sites (−Cu–O–Cu−). Importantly, no plasmon resonance near 567 nm^43^ is detected, confirming the absence of metallic Cu clusters (Cu_*x*_) or larger nanoparticles under the present pretreatment conditions.

**Figure 3:**
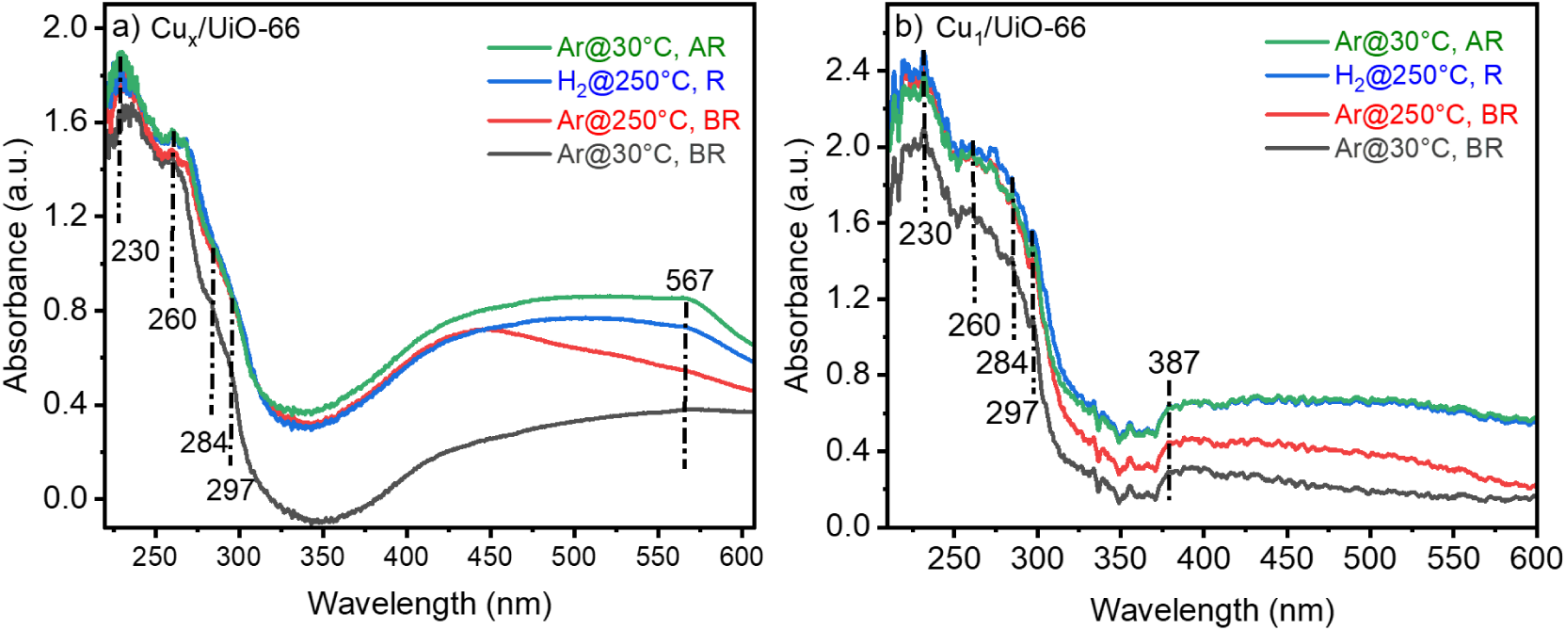
In situ UV–vis spectra recorded for Cu_*x*_/UiO-66 (a) and Cu_1_/UiO-66 (b) catalysts under different conditions: Ar at 30 °C (black line); Ar at 250 °C (red); 10 vol % H_2_/Ar at 250 °C after 2 h of exposure (green); and Ar at 30 °C (blue line) again.

### 3.3 Quantum Chemical Modeling and Spectral Interpretation

Building on the experimental analysis, we employed TD-DFT simulations of Cu_1_-MOF, bare Cu clusters (Cu_*x*_), and Cu_3_-MOF to examine how structural, electronic, and computational factors shape the UV–vis absorption profiles. Direct comparison with in situ spectra pinpoints configurations that best match the experimental fingerprints of Cu_1_/UiO-66 and Cu_*x*_/UiO-66, providing molecular-level insight into the identity and evolution of the active sites. These findings are detailed in the following subsections.

#### 3.3.1 Spectral Signatures of Cu_1_-MOF Models

Unless otherwise specified, all Cu_1_-MOF results refer to the Cu­(I) oxidation state, with acetate ligands used as surrogates for the native UiO-66 linkers. In this configuration, CAM-B3LYP/Def2SVPP calculations predict six distinct absorption peaks at 221, 253, 277, 344, 373, and 455 nm, with the most intense feature at 221 nm and a moderately strong band at 373 nm (see Figures 4a, S3 and S4, as well as our previous work.^22^ The high-energy peaks (221, 253, and 277 nm) arise from a combination of Cu-centered d–d excitations, Cu → Zr MMCT, and Cu → carboxylate MLCT transitions, enabled by electronic coupling between the Cu­(I) 3d orbitals and low-lying empty orbitals on Zr or ligand π* orbitals. The 344 nm feature is dominated by Cu-centered d–d transitions with minor contributions from the Zr_6_O_8_ node and linkers to Cu, while the lower-energy bands at 373 and 455 nm correspond to additional Cu-based d–d excitations. Although d–d transitions are formally forbidden for the closed-shell 3d^10^ configuration of isolated Cu­(I), ligand-field perturbations within the MOF relax these restrictions, producing measurable intensity and shifting the transitions to lower energies relative to typical Cu­(II) systems. Overall, the simulated spectrum reflects a complex interplay of charge-transfer and symmetry-allowed d–d excitations shaped by the unique Cu­(I) coordination environment in the Zr_6_O_8_ node.

**Figure 4:**
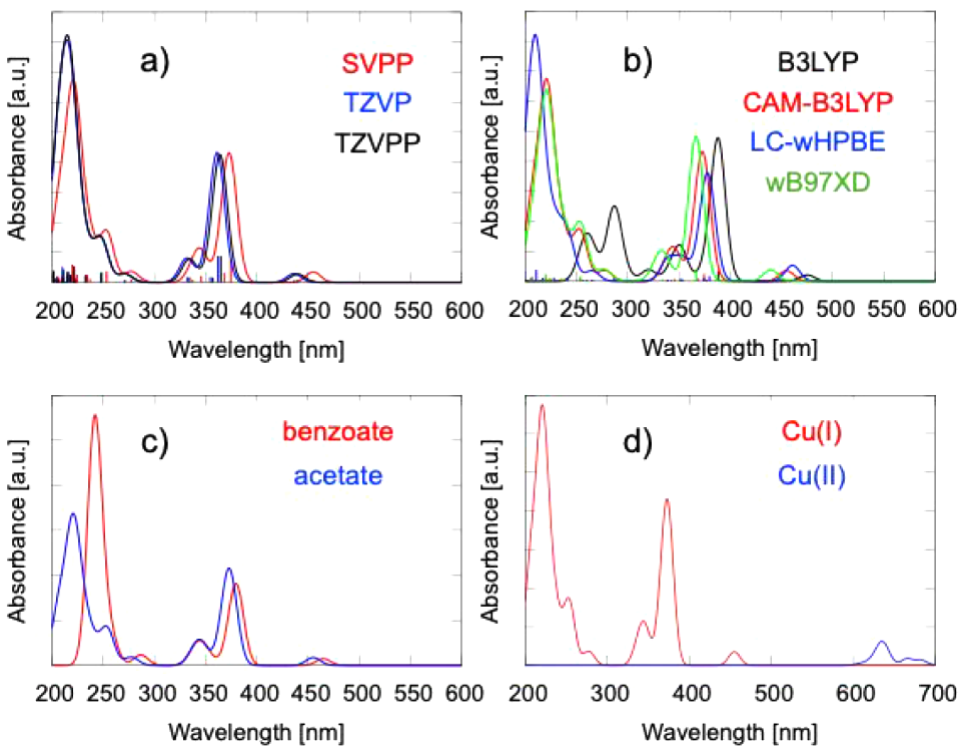
Calculated UV–vis spectra of the Cu_1_-MOF model, representing the single-site Cu_1_/UiO-66 catalyst with an acetate ligand and a monovalent Cu­(I) center. Spectra are shown for: (a) CAM-B3LYP functional with three basis sets (Def2SVPP, Def2TZVP, and Def2TZVPP); (b) Def2SVPP basis set combined with different functionals (B3LYP, CAM-B3LYP, LC-ωHPBE, and ωB97XD); (c) CAM-B3LYP/Def2SVPP applied to Cu_1_–MOF models with either acetate or benzoate ligands, both containing Cu­(I); and (d) Cu­(I) and Cu­(II) oxidation states, each with acetate ligands. All spectra use a Gaussian broadening of 10 nm (half-width). For comparison with a broader convolution (40 nm) (see Figures S3–S5).

##### 3.3.1.1 Basis Set Effects

The effect of the basis set size on the predicted UV–vis spectra was evaluated using the CAM-B3LYP functional with the Def2SVPP, Def2TZVP, and Def2TZVPP basis sets. The Def2TZVP spectrum closely matches that obtained with Def2SVPP, differing only by minor blue shifts, with peaks appearing at 215, 246, 270, 332, 361, and 437 nm (Figure 4a). These small shifts reflect the greater flexibility of the triple-ζ basis in describing the electronic transitions. The Def2TZVPP spectrum is virtually identical to that of Def2TZVP, showing a maximum wavelength difference of ∼3 nm (215, 246, 271, 334, 364, and 440 nm) and negligible changes in relative intensities. Although Def2TZVPP, with its additional polarization functions, can offer higher accuracy for resolving subtle spectral features, the overall profiles are consistent across all three basis sets. This consistency indicates that the more compact Def2SVPP basis can still capture the key electronic transitions of the Cu_1_-MOF model, making it a computationally efficient choice for larger-scale or high-throughput simulations.

##### 3.3.1.2 DFT Functional Dependence

The influence of exchange-correlation functional choice on the predicted UV–vis spectra of the Cu_1_–MOF model was assessed using B3LYP, CAM-B3LYP, LC-ωHPBE, and ωB97XD in combination with Def2SVPP and Def2TZVP basis sets (see Figures 4b and S3–S5). With Def2SVPP, CAM-B3LYP and ωB97XD produced nearly identical profiles, each yielding six well-defined peaks spanning 221–455 nm (CAM-B3LYP) or 221–440 nm (ωB97XD). Both reproduced the relative spacing and intensities of the Cu­(I)-centered d–d, MMCT, and MLCT features. LC-ωHPBE yielded a more compressed spectrum (210, 265, 351, 378, and 461 nm), with a blue shift of high-energy bands and a red shift of the lowest-energy feature, reflecting a different balance of local and charge-transfer excitations. In contrast, B3LYP yielded a markedly red-shifted, less resolved spectrum (261, 287, 321, 351, 388, and 475 nm), reflecting its known tendency to underestimate long-range charge-transfer excitation energies and produce broader peak spacing.

Results with Def2TZVP followed the same trend (see Figure S5). CAM-B3LYP (215, 246, 270, 332, 361, 437 nm) and ωB97XD (216, 248, 270, 330, 364, 436 nm) remained in close agreement, reinforcing the compatibility of these two range-separated hybrid functionals in accurately capturing both local and long-range excitations. LC-ωHPBE showed minor deviations (205, 260, 338, 364, 441 nm), while B3LYP again yielded red-shifted, broadened features (258, 277, 308, 340, 379, and 460 nm), consistent with its limitations for long-range charge-transfer transitions.

##### 3.3.1.3 Ligand Effects

Substituting the acetate ligand in the Cu_1_-MOF model with a benzoate linker produces a red-shifted UV–vis spectrum (CAM-B3LYP/Def2SVPP) with fewer but well-resolved transitions. Five main peaks appear at 242, 287, 345, 380, and 465 nm, dominated by the intense 242 nm band (Figure 4c). This shift and peak reduction stem from the benzoate’s extended π-conjugation, which increases electronic delocalization and strengthens LMCT and intraligand π→π* excitations. Detailed assignments are provided in Note S1, Figures S6 and S7. These results highlight the pronounced sensitivity of Cu­(I) excited states to the electronic nature of coordinating ligands and the pivotal role of π-conjugation in tuning the optical properties of MOF-supported copper catalysts, providing a plausible explanation for the diverse UV–vis features of Cu­(I) species reported in the literature, particularly those arising from variations in ligand environments.

##### 3.3.1.4 Oxidation-State Effects

Substituting Cu­(I) with Cu­(II) in the Cu_1_-MOF model induces a pronounced red-shift, producing eight absorption bands at 636, 667, 682, 721, 759, 832, 882, and 977 nm (CAM-B3LYP/Def2SVPP), all within the visible–NIR range (Figure 4d). The Cu­(II) spectrum is dominated by a single peak at 882 nm, with the remaining features appearing as weak, broad bands. This shift reflects the open-shell d^9^ configuration of Cu­(II), which enables spin- and symmetry-allowed d–d transitions and weak ligand-field excitations extending beyond the UV. The stark contrast between the sharp, UV-localized Cu­(I) spectrum and the broad, NIR-shifted Cu­(II) profile underscores the strong influence of oxidation state on the excitation landscape, highlighting UV–vis spectroscopy as a sensitive probe for oxidation-state discrimination in Cu-based MOF catalysts. Similar qualitative trends with LC-ωHPBE and ωB97XD further support the robustness of this conclusion (see Figure S8).

In summary, Cu­(I)-based models display absorption bands almost entirely below 400 nm, consistent with a closed-shell d^10^ configuration in a weak ligand field, whereas Cu­(II)-based models exhibit additional low-energy features from spin-allowed d–d and charge-transfer transitions extending broadly from ∼450 to 980 nm. These visible-NIR features are absent in experimental spectra recorded under reducing conditions, which consistently show strong absorption only below 350–370 nm and minimal intensity above 450 nm, confirming Cu­(I) as the predominant oxidation state under inert or mildly reducing atmospheres. The agreement between these experimental profiles and TD-DFT predictions for Cu­(I)–acetate/benzoate models, particularly when CAM-B3LYP or ωB97XD is used with triple-ζ basis sets, underscores the reliability of these computational setups for reproducing experimentally relevant electronic transitions. From this comparison, we recommend: (i) Cu­(I) as the predominant oxidation state under such conditions; (ii) benzoate linkers for enhanced LMCT transitions and extended UV coverage, with acetate linkers as a computationally efficient alternative that preserves the main spectral features; and (iii) CAM-B3LYP or ωB97XD with Def2TZVPP for the highest accuracy or CAM-B3LYP with Def2SVPP and acetate linkers for large-scale or high-throughput simulations.

#### 3.3.2 Spectral Signatures of Cu_*x*_ Models

We computed the UV–vis absorption spectra of bare copper clusters (Cu_*x*_, *x* = 2–8) employing the CAM-B3LYP functional with Def2SVPP, Def2TZVP, Def2TZVPP, and Def2QZVP basis sets to examine the influence of cluster nuclearity on their optical characteristics. At the CAM-B3LYP/Def2SVPP level, all of the clusters exhibit distinct and well-resolved absorption bands (Figure 5). The Cu_2_ spectrum features two prominent bands centered near 307 and 470 nm, with weaker transitions at 218 and 547 nm. Increasing the cluster size leads to red-shifting and spectral complexity from Cu_3_ to Cu_8_ (Figure 5). For Cu_3_, absorption peaks shift deeper into the visible region, featuring intense bands at 439, 470, and 513 nm and a subtle shoulder at 540 nm, indicative of the emerging Cu–Cu electronic delocalization and charge-transfer excitations. The Cu_4_ cluster displays strong transitions between 358 and 423 nm, reflecting multicenter bonding development. The Cu_5_ spectrum broadens substantially, extending into the near-infrared (∼895 nm), with intense absorptions at 597 and 629 nm, signaling increased electron delocalization. From Cu_6_ through Cu_8_, the spectra become broader and more complex, showing intense collective excitations consistent with quasi-metallic behavior, including plasmon-like features. Notably, the Cu_8_ cluster exhibits a broad spectral profile spanning the UV–NIR region, dominated by a peak at 381 nm and significant absorption beyond 500 nm.

**Figure 5:**
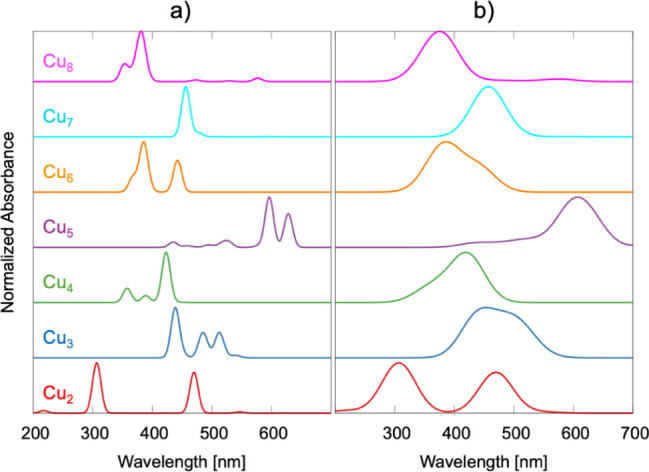
Calculated UV–vis spectra of the modeled bare Cu_*x*_ system at the CAM-B3LYP/Def2SVPP level of theory: (a) spectra with Gaussian broadening (10 nm half-width), and (b) spectra with a broader 40 nm half-width.

Importantly, odd-numbered Cu_*x*_ clusters (*x* = 3, 5, 7) generally exhibit more pronounced red-shifts than even-numbered clusters (*x* = 2, 4, 6, 8), although some spectral overlap occurs between certain odd and even cases (see Figures 5 and S9). This behavior arises from the presence of an unpaired electron in odd-numbered clusters, which induces spin polarization and enhances Cu–Cu orbital overlap, thereby increasing electronic delocalization. In contrast, even-numbered clusters possess paired electrons that favor more localized electronic states, resulting in comparatively blue-shifted absorption bands.

##### 3.3.2.1 Basis Set Effects

With Def2TZVP, the nuclearity-dependent trend remains evident: small clusters retain sharp transitions (e.g., Cu_2_ at 276 and 425 nm), while larger clusters exhibit progressively red-shifted and broadened features (Figures S10 and S11). Def2TZVP introduces a modest blue shift and improves resolution, particularly for Cu_4_ and Cu_6_, where closely spaced transitions become more distinct due to the additional polarization functions enhancing excited-state descriptions. The Def2TZVPP basis set closely reproduces Def2TZVP results but offers slightly finer resolution with better peak separation and intensity differentiation, especially in Cu_6_–Cu_8_ clusters, where greater electronic delocalization increases sensitivity to basis-set completeness. Transitioning to the quadruple-ζ Def2QZVP basis yields spectra in excellent agreement with Def2TZVPP, with only minor shifts in transition energies (e.g., Cu_2_: 280 and 431 nm) and preservation of dominant features in larger clusters (e.g., Cu_6_: 376–430 nm; Cu_8_: 373–401 nm). The changes are limited to subtle refinements in peak sharpness, symmetry, and intensity definition, particularly for intense visible-region bands, confirming that spectroscopic signatures converge at the triple-ζ level and that Def2QZVP primarily serves as a validation of basis-set completeness.

Together, these results clearly demonstrate that cluster nuclearity, rather than basis set size, governs the evolution of the UV–vis spectra in Cu_*x*_ clusters. As *x* increases, the optical response shifts from localized molecular transitions (Cu_2_–Cu_4_) to broad, intense bands (Cu_5_–Cu_8_) associated with metal-like delocalization and charge-transfer excitations. Pronounced odd–even effects, arising from spin-state differences and the presence of unpaired electrons in odd-numbered clusters, also play a key role in modulating the spectral features. While increasing the basis set from Def2SVPP to Def2QZVP leads to incremental gains in spectral resolution, the nuclearity-driven trends and characteristic spectral fingerprints remain largely unaffected.

Comparison of the in situ measured DR–UV–vis spectra on the Cu_*x*_/UiO-66 catalyst with calculated Cu_*x*_ spectra suggests that real Cu/UiO-66 catalysts likely comprise a distribution of clusters with varying nuclearities rather than a single uniform species. Among these, Cu_3_ best reproduces the experimental features observed under a reductive gas atmosphere, notably the visible-region absorption near 450–550 nm, which coincides with the emergence of plasmonic Cu bands after H_2_ treatment. Cu_5_ clusters contributed transitions in the 435–630 nm range that could account for the visible shoulder in experimental spectra; however, their pronounced ∼895 nm NIR absorption, absent experimentally, suggests they are minor or less stable species under catalytic conditions. By contrast, Cu_2_ shows no transitions beyond 300 nm, matching spectra obtained under oxidizing conditions, while larger Cu clusters (Cu_6_–Cu_8_) yield overly intense, broadened visible–NIR features inconsistent with experimental data. Overall, these results identify Cu_3_ as the most representative model for the electronic structure and optical response of small Cu clusters in UiO-66 under reducing conditions, while acknowledging the likely coexistence of multiple nuclearities with Cu_3_-like motifs dominating.

#### 3.3.3 Spectral Signatures of Cu_3_-MOF Models

To elucidate the electronic excitation behavior of copper clusters embedded within the UiO-66 framework (Cu_*x*_/UiO-66), we analyzed the calculated UV–vis absorption spectra of five representative Cu_3_-MOF configurations. These models span a range of oxidation states and spin multiplicities, designed to reflect chemically plausible species under catalytically relevant redox conditions. The set includes two fully reduced, neutral models: 3Cu^0^ with spin multiplicities of 2 (M2) and 4 (M4); one fully oxidized trimer, 3Cu­(I), with total charge 0 and multiplicity (M) = 1; and two mixed-valence configurations: 2Cu^0^ + 1Cu­(I) (neutral, *M* = 1) and 2Cu­(I) + 1Cu^0^ (neutral, *M* = 2).

At the CAM-B3LYP/Def2SVPP level of theory, the neutral Cu_3_ model with a doublet spin state (3Cu^0^ M2) exhibits intense and structured absorption features spanning the visible to NIR region (see Figure 6a–b and our previous work).^22^ The spectrum shows dominant bands at 476, 524, 630, 732, and 808 nm, with the most intense peak at 630 nm closely matching the experimental UV–vis data. In the visible region, well-resolved transitions near 470, 491, 526, and 546 nm give rise to two distinct maxima at ∼476 and ∼530 nm, respectively. The peak at 476 nm is attributed primarily to MMCT transitions between Cu atoms within the trimeric cluster, with additional contributions from Cu→Zr interactions across the Zr_6_O_8_ node. The second prominent feature at ∼530 nm, associated with transitions at 526 and 546 nm, is linked to a combination of Cu→Zr and Cu→carboxylate ligand transitions, indicative of MLCT contributions coupled with a continued MMCT character. These findings highlight the complex interplay between Cu nuclearity and the MOF coordination environment in modulating the electronic structure of reduced Cu_3_ clusters.

**Figure 6:**
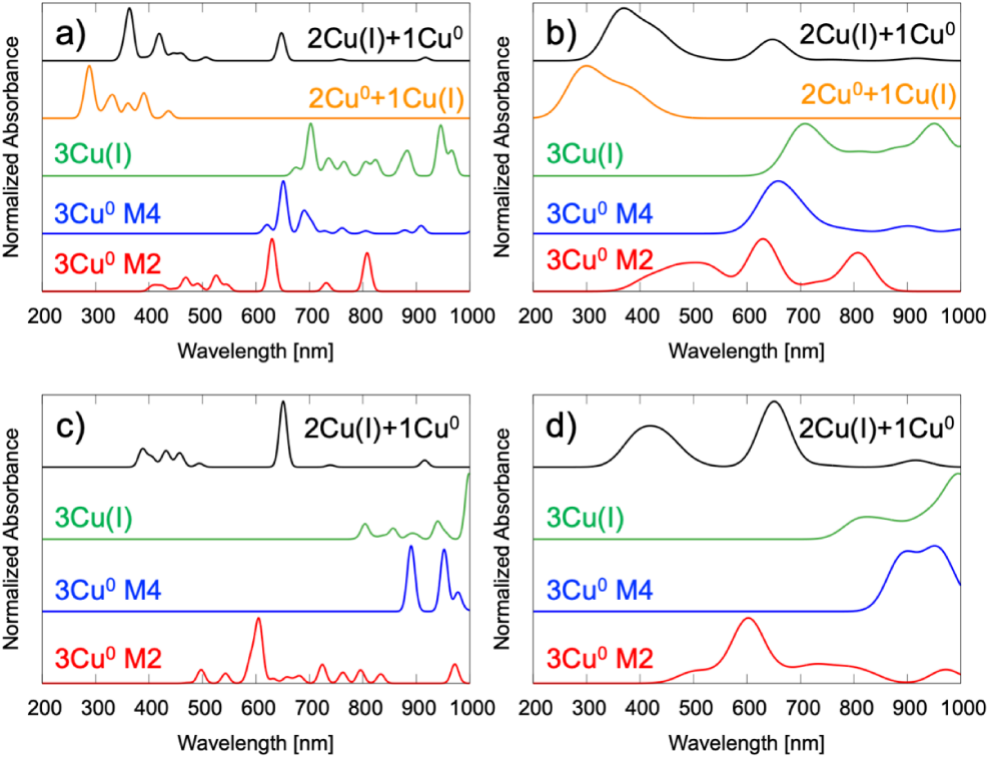
Calculated UV–vis spectra of the modeled Cu_3_-MOF system, representing various configurations at the CAM-B3LYP/Def2SVPP level: 3Cu^0^ (M2 and M4), 3Cu­(I), 2Cu­(I) + 1Cu^0^, and 2Cu^0^ + 1Cu­(I). Panels (a,b) correspond to structures with acetate linkers, while panels (c,d) correspond to benzoate linkers. Gaussian broadening of 10 nm half-width is applied in (a,c) and 40 nm in (b,d).

The high-spin variant (3Cu^0^ M4), while retaining similar redox character, exhibits broader, red-shifted transitions extending from ∼620 to 910 nm (see Figure 6a–b). This shift reflects a spin-state dependence in the electronic delocalization across the Cu_3_ cluster, consistent with the enhanced stabilization of low-energy excited states and increased delocalization in the high-spin Cu_3_ core. In contrast, the UV–vis spectrum of the fully oxidized 3Cu­(I)-MOF model exhibits multiple low-energy absorption features spanning 675–966 nm (Figure 6a–b). These features arise exclusively from transitions into the LUMO, with two dominant peaks at 703 and 946 nm corresponding to LMCT from the carboxylate groups to the Cu_3_ cluster and Zr ions, π→π* contributions, and d–d excitations within the Cu_3_ cluster. These results highlight the key role of multinuclear copper centers and linker electronic structure in modulating the optical properties of MOF-based systems. Detailed assignments are provided in Note S2 and Figure S12.

The mixed-valence 2Cu^0^ + 1Cu (I) model exhibits a distinct UV–vis fingerprint with intense transitions in the near-UV to visible range, marked by peaks at 289, 332, 361, and 391 nm and a sharp intensity drop beyond 450 nm (Figure 6a–b). These features reflect partially delocalized Cu–Cu and Cu–ligand excitations, indicating strong electronic communication within the trinuclear Cu cluster and its coupling to the linker. In contrast, the 2Cu­(I) + 1Cu^0^ configuration shows a broader, less intense profile spanning 360–920 nm, with main bands at 364, 420, and 648 nm, and additional weaker features at 447, 460, 507, 758, and 918 nm (Figure 6a–b). The intense 364 nm band originates mainly from LMCT and π→π* excitations with minor MMCT character, while the 420 and 648 nm bands are dominated by MMCT within the Cu_3_ cluster with LMCT, MLCT, and π→π* contributions. These results underscore the significant delocalization and linker coupling in mixed-valence Cu_3_ clusters, highlighting their role in tuning the photophysical properties of MOF-based systems. Detailed assignments are provided in Note S3, Figures S13 and S14.

##### 3.3.3.1 Ligand Effects

The effect of ligand identity on the Cu_3_-MOF optical response was examined by replacing acetate with benzoate linkers in four configurations (Figure 6c–d). In 3Cu^0^ M2, benzoate retains the overall spectral profile but broadens and red-shifts bands to 497, 543, 605, 724, and 796 nm, with enhanced 650–800 nm intensity due to greater delocalization and stabilized charge-transfer states from its extended π-system. Comparable trends are observed for 3Cu^0^ M4, 3Cu­(I), and 2Cu­(I) + 1Cu^0^: the high-spin 3Cu^0^ M4 shows deeper NIR absorptions (891, 953, 978 nm), 3Cu­(I) displays CT-enhanced bands at 804–999 nm, and 2Cu­(I) + 1Cu^0^ presents sharper visible–NIR peaks at 388–651 nm. Overall, benzoate substitution consistently red-shifts, broadens, and intensifies low-energy transitions, reflecting stronger electronic coupling and enhanced delocalization. For details, see Note S4.

##### 3.3.3.2 Basis Set and DFT Functional Effects

The impact of the basis set and DFT functional on the optical properties of Cu_3_-MOF was assessed through UV–vis spectral comparisons (see Note S5 and Figure S15). Increasing the basis set size from Def2SVPP to larger triple-ζ sets (Def2TZVP, Def2TZVPP) produced only minor blue shifts without altering peak number, spacing, or relative intensities. While the enhanced polarization and delocalization treatment marginally refine excitation energies, the practical gain in spectral resolution is minimal. In contrast, the functional choice had a far greater impact: M06L and B3LYP yielded markedly red-shifted, broadened spectra with diminished band separation, reflecting their limited ability to describe Cu–Cu interactions and long-range LMCT excitations. CAM-B3LYP, by comparison, produced sharper, more resolved features, underscoring the advantage of range-separated hybrid functionals for accurately capturing the excited-state electronic structure of Cu_3_-MOF systems.

##### 3.3.3.3 Comparison with Experiment

Correlating the calculated and experimental UV–vis spectra suggests that several Cu_3_-MOF configurations can account for the spectral evolution of Cu_*x*_/UiO-66 before and after H_2_ reduction. Prior to reduction, the experimental bands at 230, 260, 284, and 297 nm closely resemble the predicted UV transitions of mixed-valence clusters, 2Cu^0^ + 1Cu­(I) and 2Cu­(I) + 1Cu^0^, which display strong absorptions in the 250–450 nm range. The absence of visible-range features beyond 300 nm agrees with the lack of metallic Cu clusters in the as-synthesized material. Upon H_2_ reduction, the appearance of a broad visible band centered around 567 nm, characteristic of plasmon resonance, together with persistent UV absorptions, aligns with contributions from neutral 3Cu^0^ M2 clusters and partially reduced mixed-valence species. While no single Cu_3_–MOF model fully reproduces the experimental spectra, the calculated results provide a mechanistic picture linking the oxidation state, nuclearity, and coordination environment to the evolving optical response under reductive conditions.

While the qualitative trends discussed above and in the previous subsections highlight plausible oxidation states and nuclearities, a more rigorous evaluation requires quantitative comparison between theory and experiment. In the following section, we apply two complementary correlation approaches: (A) matching each experimental spectrum to its closest single calculated model, and (B) reconstructing the experimental spectra from multimodel linear combinations, enabling a more nuanced interpretation of the species distribution.

#### 3.3.4 Theory–Experiment Quantitative Correlation

##### 3.3.4.1 Single-Model Correlation

To quantitatively evaluate the agreement between theory and experiment, we performed correlation analyses between calculated and experimental UV–vis spectra using a one-to-one (1:1) matching approach. Each calculated spectrum, broadened with Gaussian functions at half-widths of 10, 20, 30, 40, and 50 nm, was individually compared to the corresponding in situ spectrum. Pearson correlation coefficients were computed to quantify the spectral similarity. This procedure enables direct identification of the single theoretical model that best reproduces the experimental spectrum under a given condition.

For the Cu_1_/UiO-66 catalyst (Cu­(I) oxidation state), spectra calculated with B3LYP/Def2SVPP showed poor agreement with the experiment, with correlation coefficients increasing only modestly from 0.22 (10 nm) to 0.30 (30 nm). In contrast, range-separated hybrid functionals—CAM-B3LYP, ωB97XD, and LC-ωHPBE—demonstrated significantly improved correlations. At 30 nm broadening, both CAM-B3LYP and ωB97XD achieved strong agreement (correlation ≈ 0.79), while LC-ωHPBE gave a slightly lower correlation of 0.75.

Replacing acetate with benzoate ligands in the Cu_1_-MOF model (CAM-B3LYP/Def2SVPP) slightly improved the correlation to 0.83, underscoring the role of ligand π-conjugation in capturing electronic transitions in Cu_1_/UiO-66 catalysts. This result also confirms that both acetate and benzoate can serve as suitable representative ligands for modeling the coordination environment of the experimental catalyst.

The effect of basis set choice was evaluated by comparing CAM-B3LYP spectra computed with Def2SVPP, Def2TZVP, and Def2TZVPP. All three produced virtually identical results, with correlation coefficients at 30 nm broadenings of 0.79, 0.77, and 0.78, respectively. These values indicate no significant improvement in agreement with the experiment as the basis set size increases, confirming that Def2SVPP provides comparable accuracy to the larger triple-ζ sets while offering lower computational cost.

Simulating the Cu­(II) oxidation state produced negative correlations across all tested functionals and basis sets, further reinforcing Cu­(I) as the dominant oxidation state in the Cu_1_–MOF active site under the studied conditions. This conclusion is fully consistent with previous spectroscopic evidence, including X-ray absorption, FTIR, and UV–vis, reported for Cu_1_/UiO-66 catalysts.^10,11^

For the Cu_*x*_/UiO-66 catalyst, all calculated spectra of the Cu_3_-MOF models, covering five configurations (3Cu^0^ in M2 and M4, 3Cu­(I), 2Cu^0^ + 1Cu­(I), and 2Cu­(I) + 1Cu^0^), two ligand environments (acetate and benzoate), and multiple functionals and basis sets, consistently showed negative correlations with the experimental data. This confirms that the experimental spectra under the studied conditions cannot be explained solely by the MOF-supported Cu_3_ clusters. Instead, the results indicate that the observed experimental spectral features arise from a mixture of isolated Cu single sites (Cu_1_) and Cu clusters of varying nuclearity within the MOF. In particular, Cu_1_ sites account for the strong absorption below 300 nm, while the incorporation of Cu_3_ components contributes to the broad shoulder in the 480–530 nm region observed after reduction.

##### 3.3.4.2 Multi-Model Linear Combination Correlation

To account for the heterogeneous mixture of single-site and clustered Cu species in the Cu_*x*_/UiO-66 catalysts, we modeled each experimental UV–vis spectrum as a weighted sum of TD–DFT-simulated spectra spanning multiple Cu oxidation and spin states. The reference library comprised spectra from Cu_1_-MOF, Cu_3_-MOF, and bare Cu_*x*_ clusters, including 9 Cu_3_-MOF configurations (5 acetate- and 4 benzoate-based), 3 Cu_1_-MOF configurations (2 acetate-based: Cu­(I) and Cu­(II), 1 benzoate-based Cu­(I)), and 7 bare Cu_*x*_ clusters (*x* = 2–8). Optimal, physically meaningful weighting coefficients were obtained via non-negative least-squares (NNLS) regression, ensuring all contributions remained positive. This approach yielded quantitative reconstructions of the experimental spectra and insight into the speciation and distribution of active sites under the studied conditions. Mathematically, the model is expressed as

$$A_{\exp}\left(\lambda \right)=\overset{n}{\underset{i=1}{\sum }}w_{i}\times A_{i}\left(\lambda \right)$$

where *A_exp_* (λ) is the absorbance of the experimental spectrum at wavelength λ, *A_i_* (λ) is the absorbance of the simulated i-th configuration (e.g., a specific Cu_1_-MOF, Cu_3_-MOF, or Cu_*x*_ species) at wavelength λ, *w_i_* is the regression coefficient (weight) representing the contribution of the *i*-th configuration, and n is the total number of simulated configurations included in the multilinear regression model (here, *n* = 20).

For each experimental spectrum, fits were performed at Gaussian broadenings of 10–50 nm, and agreement with the reconstructed spectra was evaluated using multiple complementary metrics. The Pearson correlation coefficient (*r*) served as the primary indicator, capturing overall shape similarity by simultaneously penalizing peak position mismatches (*x*-axis) and intensity differences (*y*-axis). Moreover, we report the normalized RMSE (nRMSE) and adjusted *R* ^2^ values (*R* ^2^_adj_). Confidence intervals for *r* (Fisher z-transform), bootstrap CIs for coefficients, and additional diagnostics (lagged and first-derivative correlations) ensured robust evaluation. Full metric definitions and calculation procedures are provided in Note S6.

For the Cu_*x*_/UiO-66 catalyst, linear regression analysis enabled quantitative deconvolution of the experimental UV–vis spectra into contributions from distinct modeled structural motifs (Figure 7a–d). High Pearson correlation coefficients were achieved under four conditions: **0.84** for EXP1 (before H_2_ reduction at 30 °C), **0.87** for EXP2 (before H_2_ reduction at 250 °C), **0.91** for EXP3 (during 2 h of H_2_ reduction), and **0.95** for EXP4 (after H_2_ reduction at 30 °C). In EXP1 (Figure 7a), the spectrum was dominated by Cu_1_-MOF (Cu­(I)) species, contributing a combined 63% (44% from the benzoate-based model and 19% from the acetate-based model). Additional contributions included Cu_3_-MOF configurations with neutral 3Cu^0^ clusters in low-spin (M2, 22%) and high-spin (M4, 6%) forms, as well as a bare Cu_3_ cluster (8%). A minor 1% contribution was attributed to the 3Cu­(I) configuration.

As the system underwent thermal and reductive treatment (EXP2–EXP4, Figure 7b–d), the contributions from Cu_3_ (17–20%) and Cu_2_ (6–8%) clusters increased, reflecting the formation and stabilization of multinuclear Cu species. Despite this shift, Cu_1_-MOF species with both acetate and benzoate ligands (46–49%) remained significant in EXP3 and EXP4, although slightly reduced compared to EXP1 and EXP2 (53–63%). This persistence confirms that the isolated Cu­(I) motifs survive even under extended reducing conditions, coexisting with the newly formed clusters.

For the Cu single-site catalyst (Cu_1_/UiO-66), linear regression analysis yielded excellent theory–experiment agreement under all four conditions, with high correlation coefficients of **0.95** (EXP1), **0.96** (EXP2), **0.97** (EXP3), and **0.97** (EXP4). In EXP1 (before H_2_ reduction at 30 °C, Figure 7e), the spectrum was dominated by Cu_1_-MOF (Cu­(I)) species, contributing 58% from the benzoate-coordinated form and 19% from the acetate-bound form. Minor contributions arose from Cu_2_ clusters (12%) and Cu_3_-MOF with 3Cu^0^ clusters in low-spin (M2, 9%) and high-spin (M4, 3%) states.

Upon thermal activation (EXP2, before H_2_ reduction at 250 °C, Figure 7f), the Cu_1_-MOF species remained dominant at 71% (48% benzoate, 23% acetate), while the emergence of Cu_2_ (18%), Cu_3_ (3%), and Cu_5_ (8%) indicated the onset of multinuclear cluster formation. Under reductive treatment (EXP3, Figure 7g), the Cu_1_-MOF fraction decreased to 53% (33% benzoate, 20% acetate), accompanied by more pronounced multinuclear contributions: Cu_3_-MOF with 3Cu^0^ clusters in M2 (18%) and M4 (3%) configurations, Cu_2_ (17%), and Cu_3_ (6%). This shift persisted in EXP4 (postreduction at 30 °C, Figure 7h), where Cu_1_-MOF species accounted for 52% (33% benzoate, 19% acetate) and were accompanied by Cu_2_ (15%), Cu_3_ (8%), 3Cu^0^ M2 (19%), M4 (3%), and 3Cu­(I) (4%) motifs.

In summary, even in a nominally single-site Cu_1_/UiO-66 catalyst, thermal and reductive treatments induce the emergence and stabilization of multinuclear Cu species (Cu_2_–Cu_5_) while retaining a substantial fraction of persistent Cu­(I) single sites (52–77%). In contrast, Cu_*x*_/UiO-66 features a more developed multinuclear component, with increasing Cu_2_–Cu_3_ cluster contributions under reduction alongside persistent Cu­(I) motifs. This dynamically coexisting multisite architecture, quantitatively resolved here for the first time via combined single- and multimodel spectral deconvolution, offers a mechanistic basis for the complex optical signatures and directly links the catalyst’s electronic structure and redox flexibility to its catalytic behavior. Notably, subnanometer Cu clusters are known to exhibit unique coordination environments and reactivity compared to larger particles, exemplified by the exceptional CO_2_-to-methanol activity of size-selected Cu_4_ clusters on Al_2_O_3_ at low partial pressures.^44^

**Figure 7:**
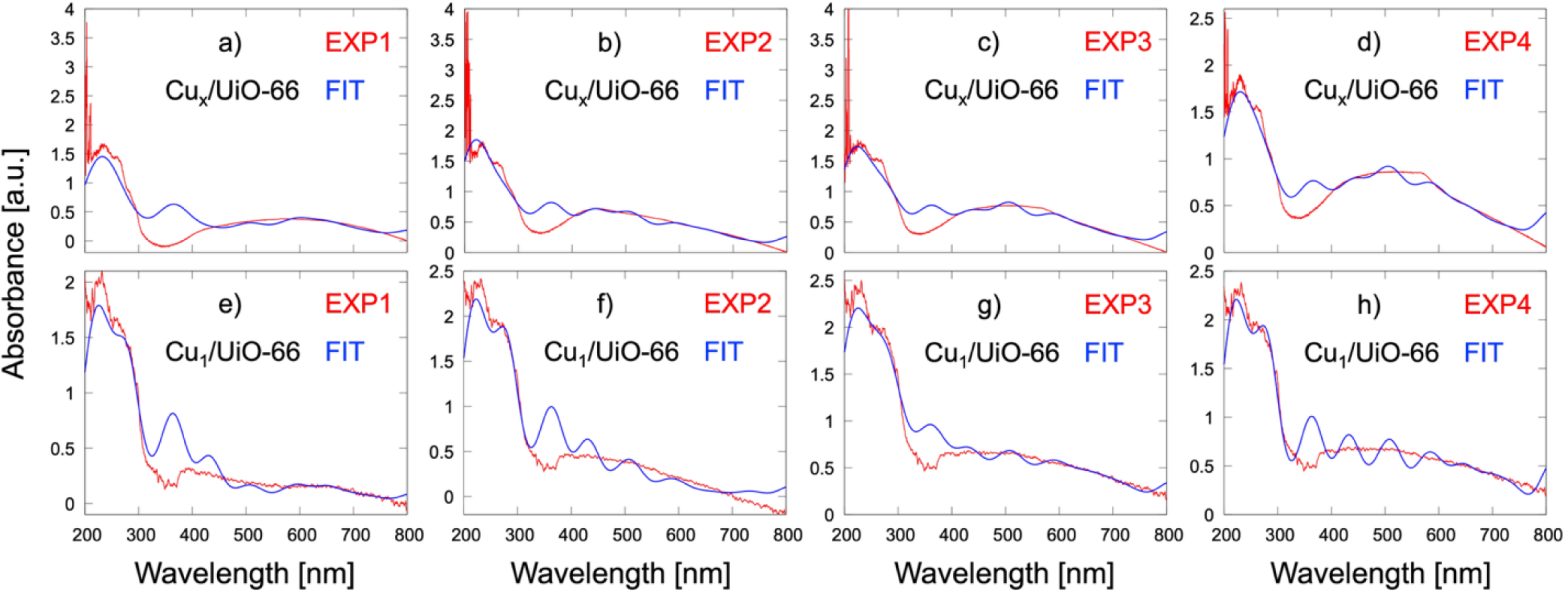
Fitted UV–vis spectra generated via multilinear regression of calculated models against experimental spectra of the Cu_*x*_/UiO-66 catalyst under various conditions: (a) EXP1 (before H_2_ reduction at 30 °C), (b) EXP2 (before H_2_ reduction at 250 °C), (c) EXP3 (during 2 h of H_2_ reduction), and (d) EXP4 (after H_2_ reduction at 30 °C). Panels (e-h) show the corresponding fits for the Cu_1_/UiO-66 catalyst. All fitted spectra were plotted using Gaussian broadening with a 40 nm half-width.

## 4 Concluding Remarks and Implications

This work establishes a robust integration of in situ DR–UV–vis spectroscopy with TD-DFT simulations to resolve the oxidation state, nuclearity, and electronic structure of Cu species in UiO-66-based catalysts under reducing conditions, relevant to catalytic hydrogenation reactions. By bridging atomistic modeling with experimental in situ spectroscopy, this study delivers a versatile platform capable of distinguishing between single-site and multinuclear Cu motifs, overcoming the limitations of advanced techniques such as TEM and EXAFS.

Prior to H_2_ exposure, absorption bands at 230–297 nm are attributed to MMCT, MLCT, and LMCT transitions involving oxidized Cu­(I) in Cu_1_–MOF configurations. These features also align with TD-DFT-predicted transitions for Cu_3_–MOF configurations, especially mixed-valence clusters (2Cu^0^ + 1Cu­(I) and 2Cu­(I) + 1Cu^0^) whose absorptions fall within 250–450 nm. Upon reduction, a broad band centered at 567 nm (480–630 nm) emerges, indicating metallic Cu cluster formation. Among these, Cu_3_ motifs, especially 3Cu^0^ M2 structures, are spectroscopically and electronically dominant.

Multilinear regression analysis confirms that no single structural model fully reproduces the experimental spectra; rather, the optical response arises from the superposition of species. In Cu_1_/UiO-66, the spectra are initially dominated by Cu_1_–MOF (77%), with smaller contributions from Cu_2_ (12%) and Cu_3_–MOF (11%). Upon thermal reduction, the Cu_1_–MOF fraction decreases to ∼52–53%, while Cu_2_ (15–17%) and Cu_3_–MOF (21–22%) contributions increase. In Cu_*x*_/UiO-66, Cu_1_–MOF remains significant (46–63%), but enhanced contributions from Cu_3_–MOF (∼28%) and bare Cu_3_ clusters (8–20%) indicate expanded nuclearity and mixed-valence states under in situ conditions.

Ligand identity further modulates optical properties: replacing acetate with π-conjugated benzoates induces red-shifted LMCT transitions, improving agreement with experiment and underscoring the critical role of the coordination environment. Benchmarking identifies CAM-B3LYP and ωB97XD with the Def2TZVPP basis as optimal for spectral prediction, while CAM-B3LYP/Def2SVPP with acetate ligands provides a cost-effective alternative for large-scale modeling.

An important implication of these findings is their relevance to defect engineering in MOFs. Structural defects in UiO-66, particularly missing-linker and missing-cluster types, create under-coordinated Zr sites terminated by −OH, H_2_O, or modulator ligands, thereby perturbing the coordination geometry and electronic environment of anchored Cu species. Such changes can shift the energies and intensities of d–d, MLCT, and MMCT transitions and may induce spectral broadening or subtle band shifts in DR–UV–vis measurements. Our calculations show that UV–vis variations arise from multiple factors, including ligand identity, node termination, oxidation and spin states (Cu^0^, Cu­(I), Cu­(II), mixed valence), embedding mode (isolated vs MOF-linked clusters), and nuclearity (single sites vs various cluster sizes). Consequently, our modeling inherently captures defect-perturbed Zr_6_O_8_ environments, although a dedicated TD–DFT study of explicit defect structures, incorporating ligand substitutions and coordination asymmetries, remains a promising route to developing defect-sensitive spectral fingerprints.

Beyond Cu, the DR–UV–vis + TD–DFT fingerprinting approach is readily transferable to other transition metals in MOFs (e.g., Fe, Co, Ni, and Mn), provided that their diagnostic transitions fall within the experimental spectral window and are sufficiently intense in diffuse reflectance. Two theoretical–technical considerations are essential: (i) performing spin-polarized TD–DFT for open-shell centers and (ii) accounting for d-electron count and multiplet structure when interpreting d–d and charge-transfer bands (see Note S7). By enabling molecular-level identification of active-site nuclearity, oxidation state, and ligand environment under working conditions, this approach provides a foundation for rational ligand and site engineering, predictive catalyst design, and extension to larger clusters or alternative MOF scaffolds. It advances structure–function understanding across catalysis, energy storage, and optoelectronics, underscoring the transformative potential of coupling in situ spectroscopy with quantum modeling for atomically precise catalyst development.

## Supplementary Material



## Notes

Includes schematic representations of the catalyst preparation process (Figures S1–S2); additional calculated UV–vis spectra and frontier orbitals (HOMOs/LUMOs) (Figures S3–S15); tables summarizing absorption peaks and intensities for the Cu_1_–MOF, Cu_3_–MOF, and bare Cu_*x*_ cluster models (Tables S1–S9); and seven supporting notes. Notes S1–S5 (with Tables S10–S12) provide representative examples of assigned electronic transitions; Note S6 (with Tables S13–S14) discusses fit quality metrics between in situ spectra and weighted TD–DFT-constructed spectra; and Note S7 addresses the universality and transferability of our combined DR–UV–vis + TD–DFT approach (PDF)
