# Supplementary material for: Fingerprinting Single and Clustered Cu Sites in Metalated MOF Catalysts via TD-DFT and In Situ Diffuse Reflectance UV–Vis Spectroscopy
Source: ACS Appl Mater Interfaces. 2025 Sep 8;17(37):52840–53. doi: 10.1021/acsami.5c10338 (PMC12447388; doi:10.1021/acsami.5c10338)
Supplement: Supplementary file 1 [file am5c10338_si_001.pdf]

Supporting Information

Fingerprinting Single and Clustered Cu Sites in Metalated MOF Catalysts via TD-DFT and in situ Diffuse Reflectance UV-Vis Spectroscopy

*Soufiane Bahou[1], Riya Sehrawat[1], Bunyarat Rungtaweevoranit[2], Ali M. Abdel-Mageed[1], and Ashour A. Ahmed[1,3]**

[1] Leibniz-Institut für Katalyse e.V. (LIKAT), Albert-Einstein-Str. 29a, 18059 Rostock, Germany

[2] National Nanotechnology Center (NANOTEC), National Science and Technology Development Agency (NSTDA), Pathum Thani 12120, Thailand

[3] University of Rostock, Institute of Physics, Albert-Einstein-Str. 23-24, Rostock, Germany

E-Mail: ashour.ahmed@uni-rostock.de

1. Supporting Figures

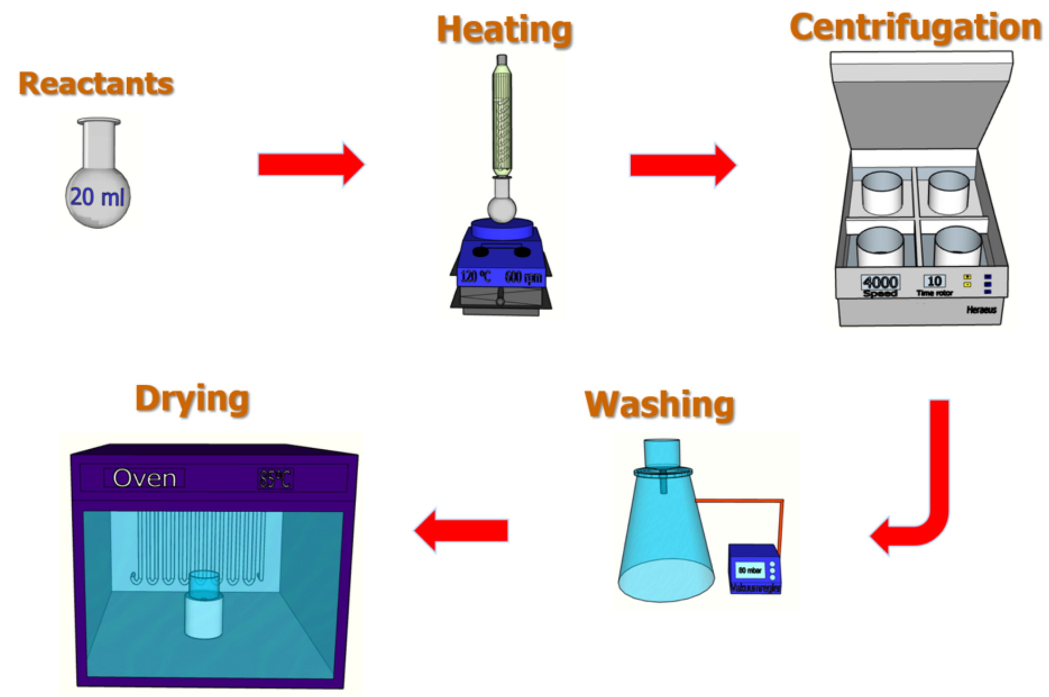

Figure S1: Schematic representation displaying the preparation process of the UiO-66.

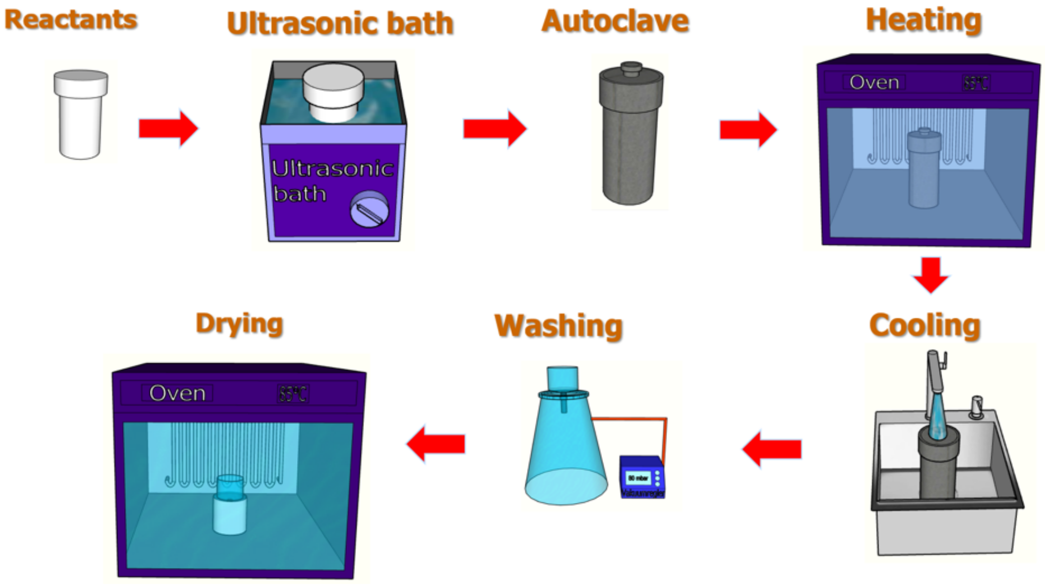

Figure S2: Schematic representation displaying the preparation process of the Cu_x/UiO-66 and Cu_1/UiO-66.

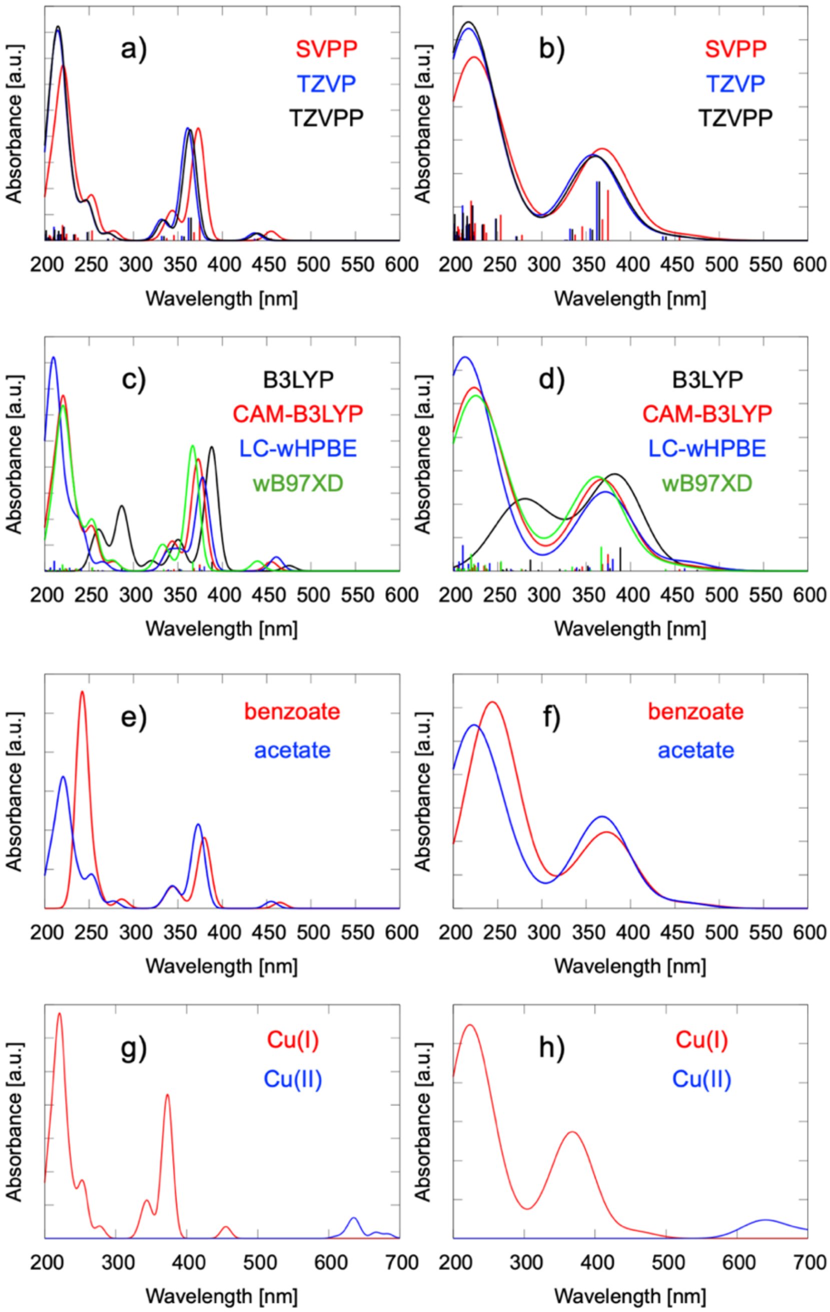

Figure S3. Calculated UV–Vis spectra of the modeled Cu_1-MOF system, representing the single-site Cu_1/UiO-66 catalyst with an acetate ligand and monovalent Cu(I) center. Spectra are shown for: (a–b) CAM-B3LYP functional with three basis sets (Def2SVPP, Def2TZVP, and Def2TZVPP); (c–d) Def2SVPP basis set combined with different functionals (B3LYP, CAM-B3LYP, LC-ωHPBE, and ωB97XD); (e–f) CAM-B3LYP/Def2SVPP applied to Cu_1-MOF models with either acetate or benzoate ligands, both containing Cu(I); and (g–h) Cu(I) and Cu(II) oxidation states, both with acetate ligands. Panels a, c, e, and g use a Gaussian broadening of 10 nm (half-width), while panels b, d, f, and h apply a broader 40 nm half-width.

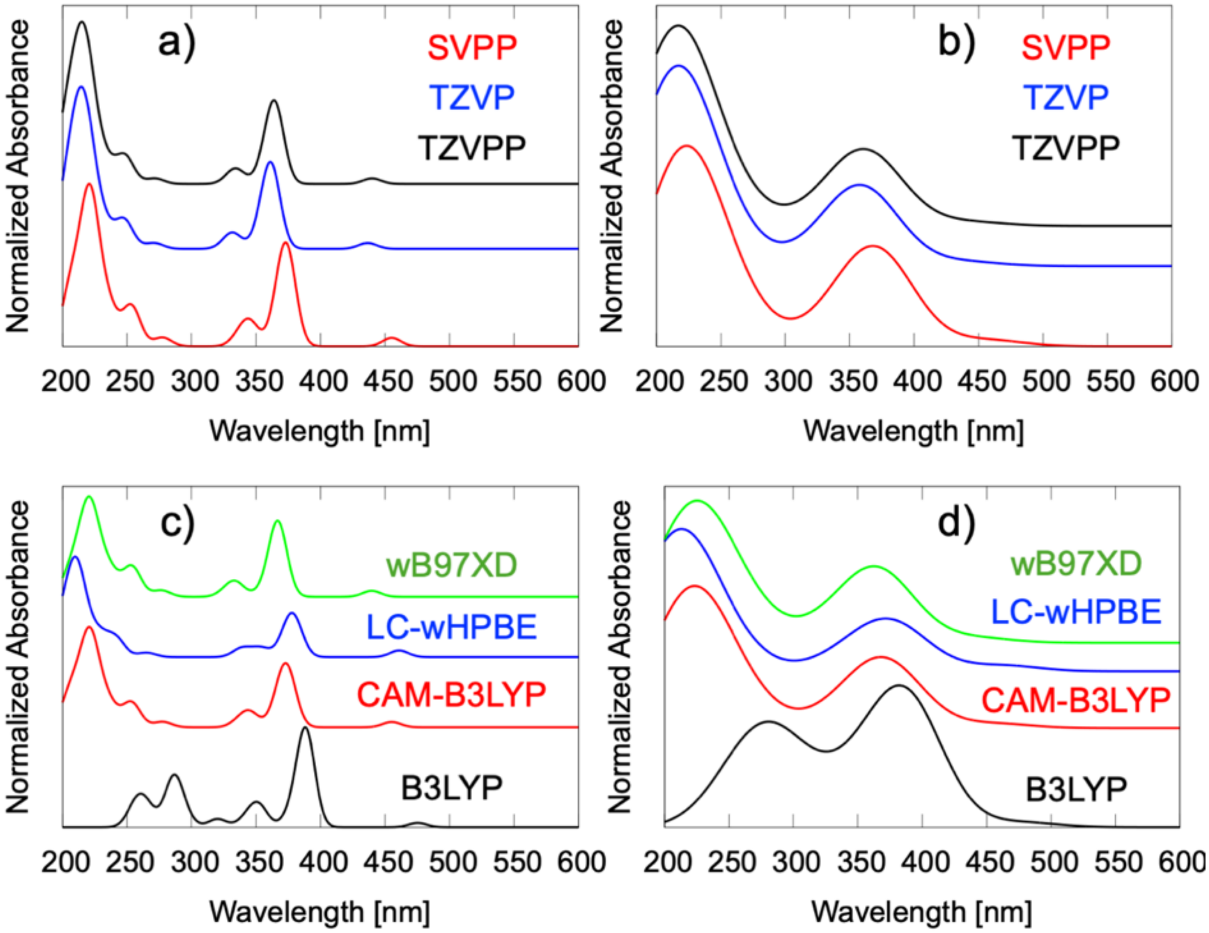

Figure S4. Calculated UV–Vis spectra of the modeled Cu_1-MOF system, representing the single-site Cu_1/UiO-66 catalyst with an acetate ligand and monovalent Cu(I) center. Spectra are shown for: (a–b) CAM-B3LYP functional with three basis sets (Def2SVPP, Def2TZVP, and Def2TZVPP); (c–d) Def2SVPP basis set combined with different functionals (B3LYP, CAM-B3LYP, LC-ωHPBE, and ωB97XD). Panels a and c use a Gaussian broadening of 10 nm (half-width), while panels b and d apply a broader 40 nm half-width.

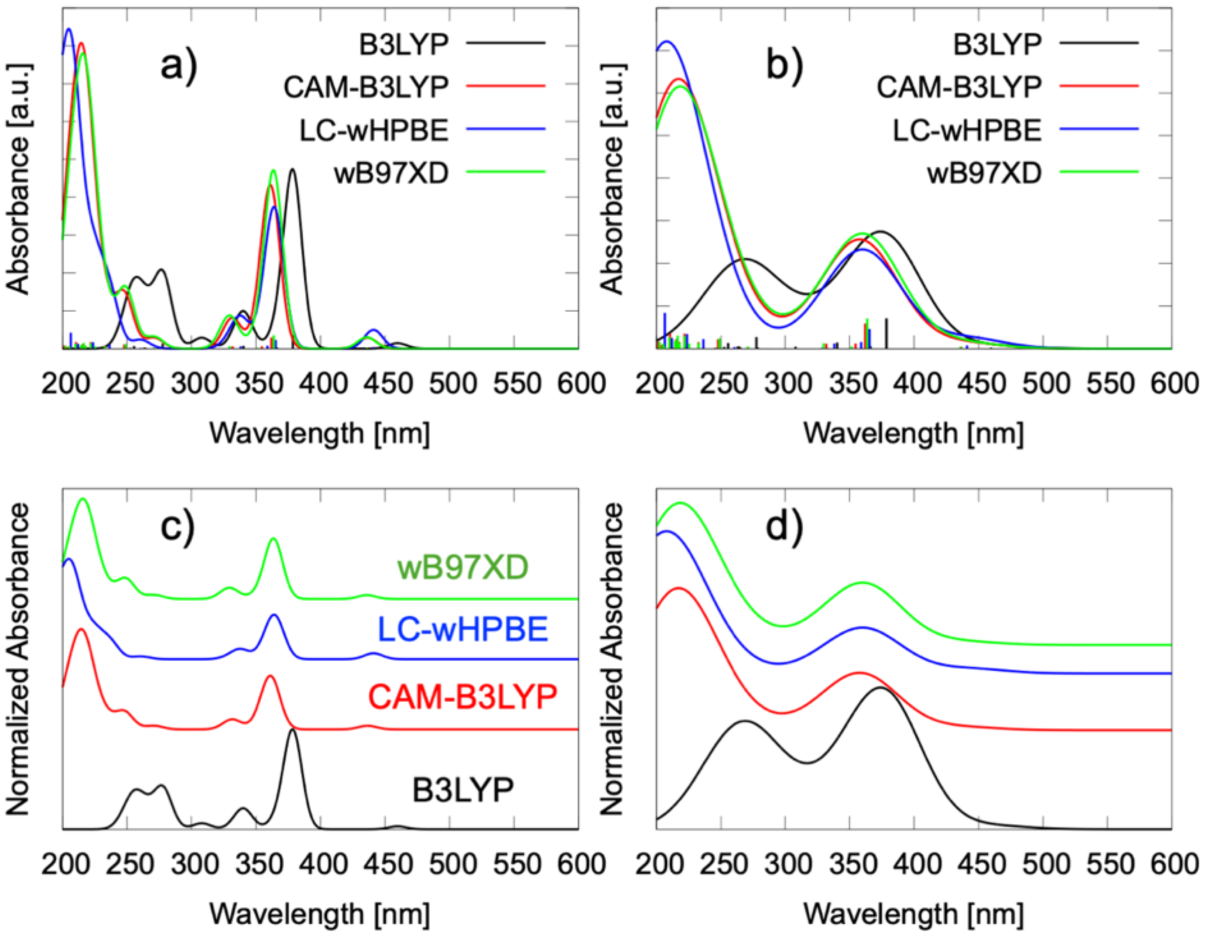

Figure S5. Calculated UV–Vis spectra of the Cu_1-MOF model, representing the single-site Cu_1/UiO-66 catalyst with an acetate ligand and a monovalent Cu(I) center, computed at the Def2TZVP basis set using various functionals (B3LYP, CAM-B3LYP, LC-ωHPBE, and ωB97XD). Panels (a) and (c) present spectra broadened with a Gaussian half-width of 10 nm, while panels (b) and (d) use a broader 40 nm half-width. Panels (a) and (c), as well as (b) and (d), are equivalent in spectral data but differ in visual presentation style.

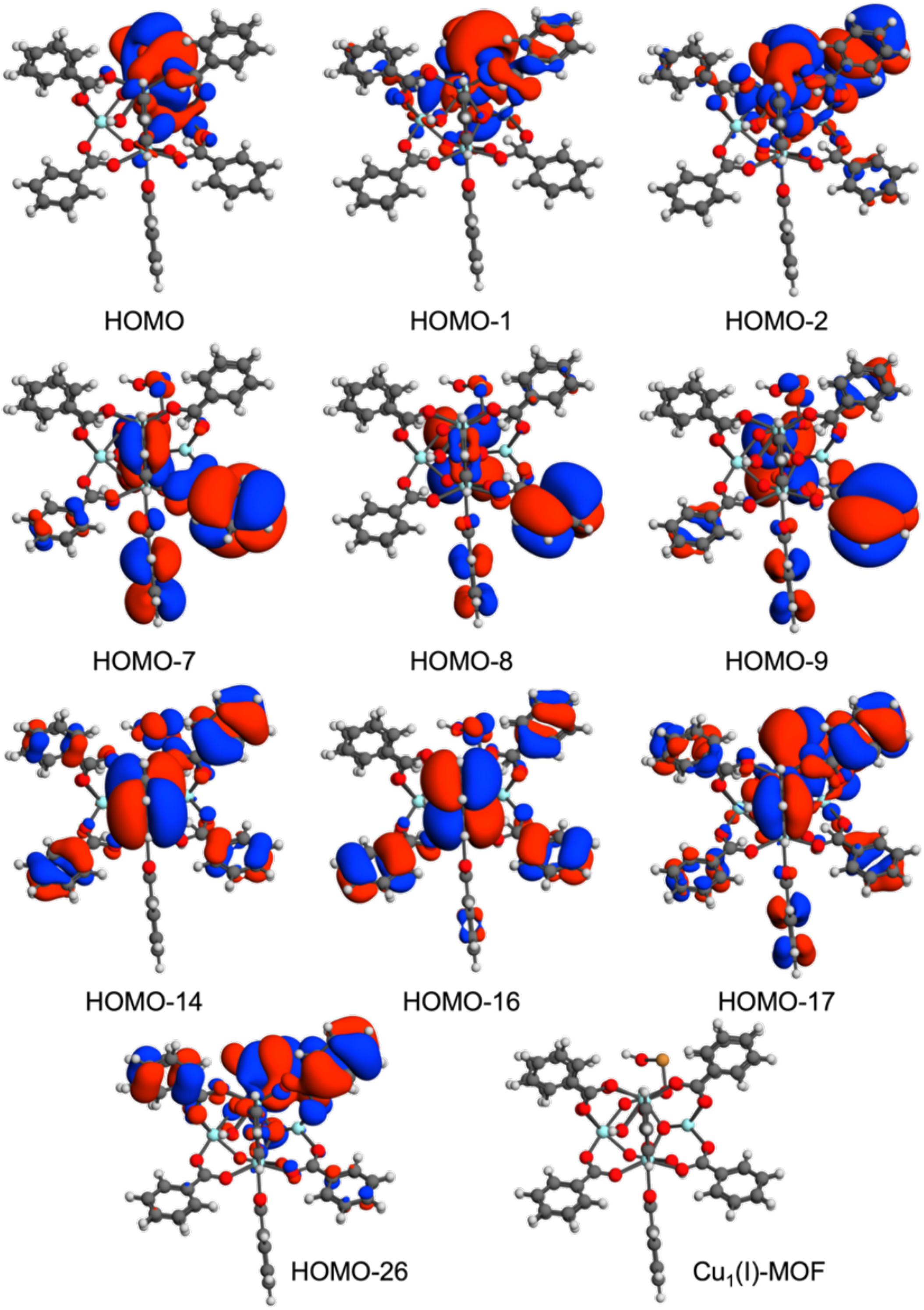

Figure S6. Geometry-optimized structure of the Cu_1-MOF model featuring an acetate ligand and a monovalent Cu(I) center, along with the dominant highest occupied molecular orbitals (HOMOs) contributing to the computed UV–Vis spectrum at the CAM-B3LYP/Def2SVPP level.

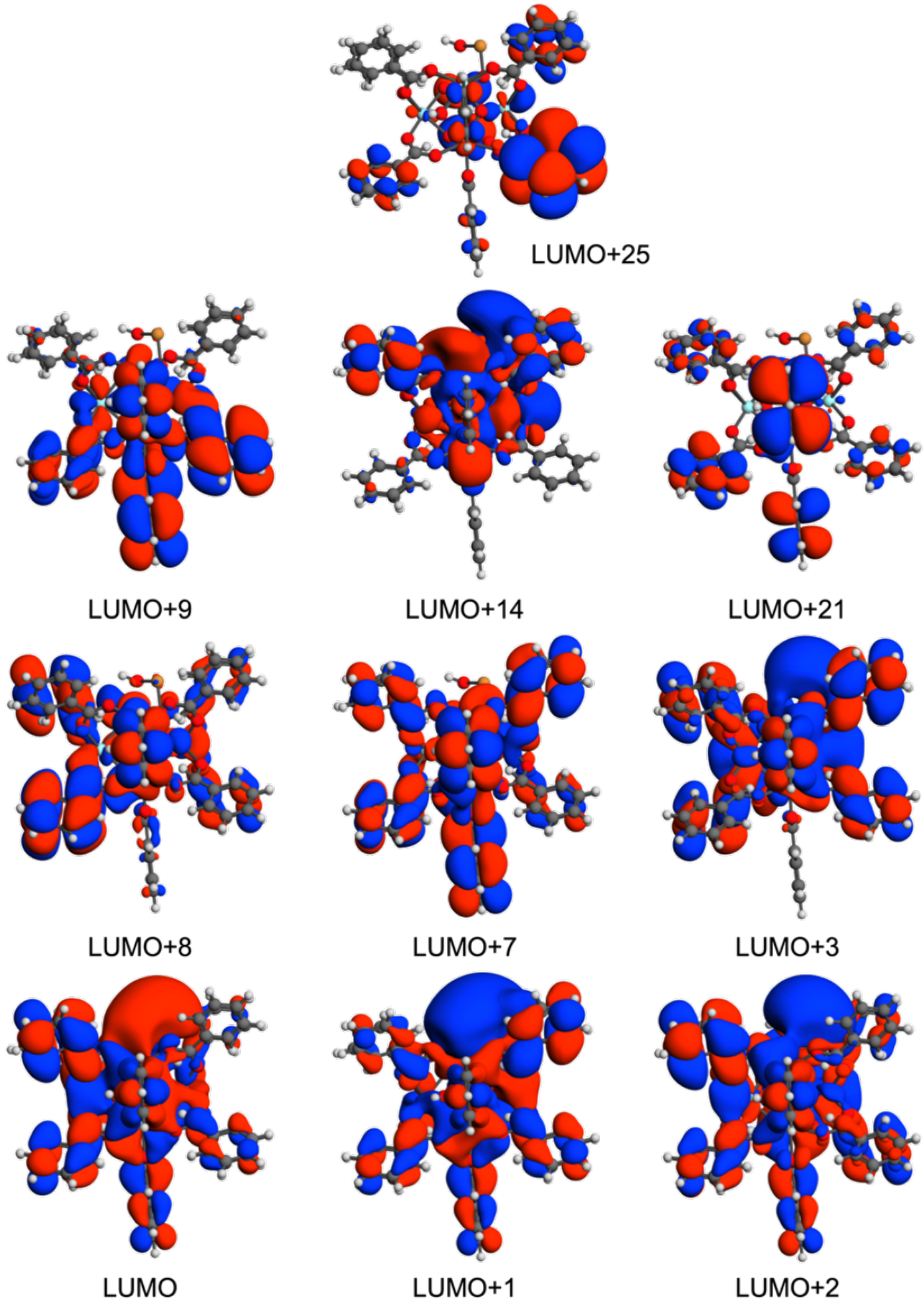

Figure S7. The dominant lowest unoccupied molecular orbitals (LUMOs) contributing to the computed UV–Vis spectrum of the Cu_1-MOF model (with acetate ligands and a monovalent Cu(I) center) at the CAM-B3LYP/Def2SVPP level.

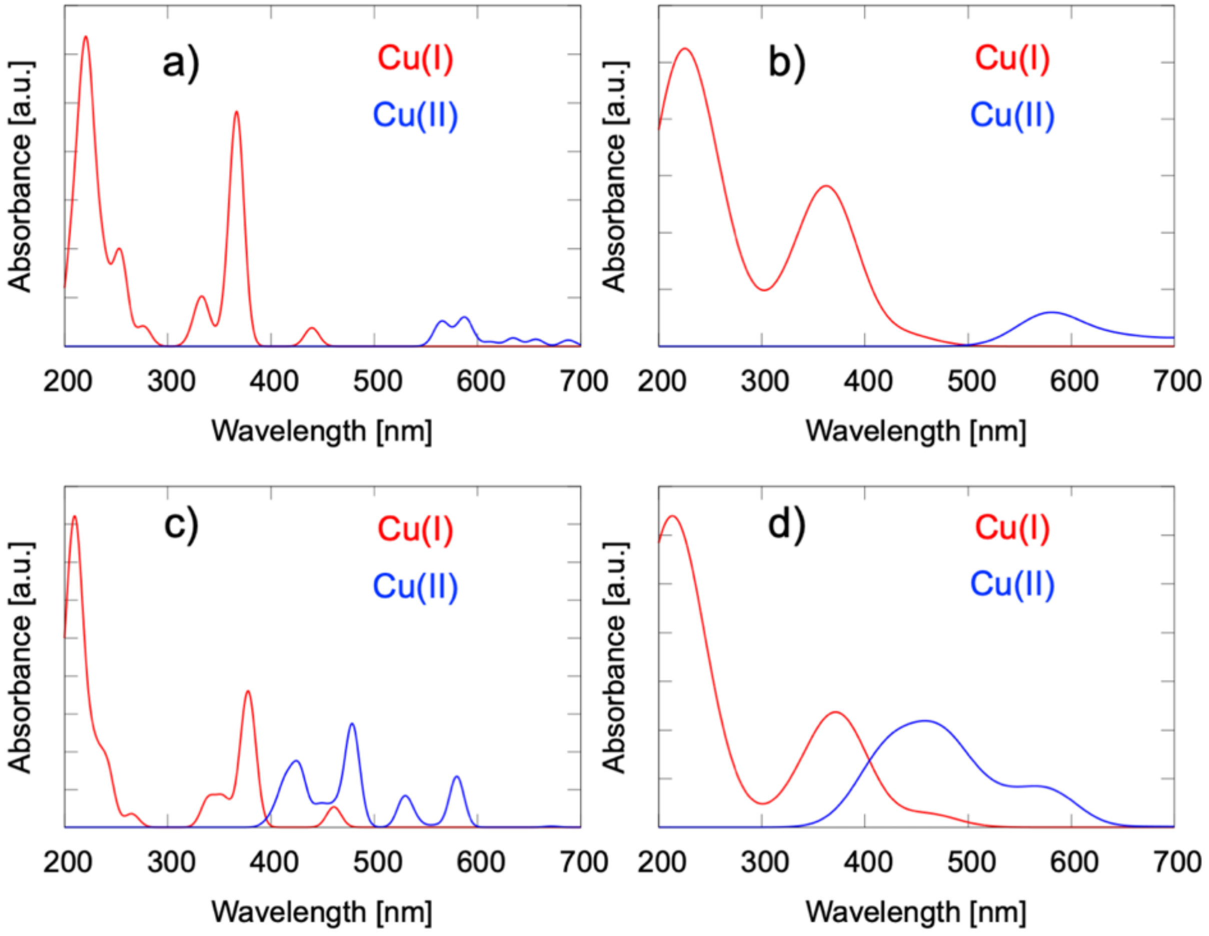

Figure S8. Calculated UV–Vis spectra of the Cu_1-MOF model with acetate ligands, comparing monovalent Cu(I) and divalent Cu(II) centers. Panels (a) and (b) show spectra computed at the ωB97XD/Def2SVPP level, while panels (c) and (d) correspond to calculations using LC-ωHPBE/Def2SVPP. Spectra in panels (a) and (c) are displayed with Gaussian broadening (10 nm half-width), whereas panels (b) and (d) apply a broader 40 nm half-width.

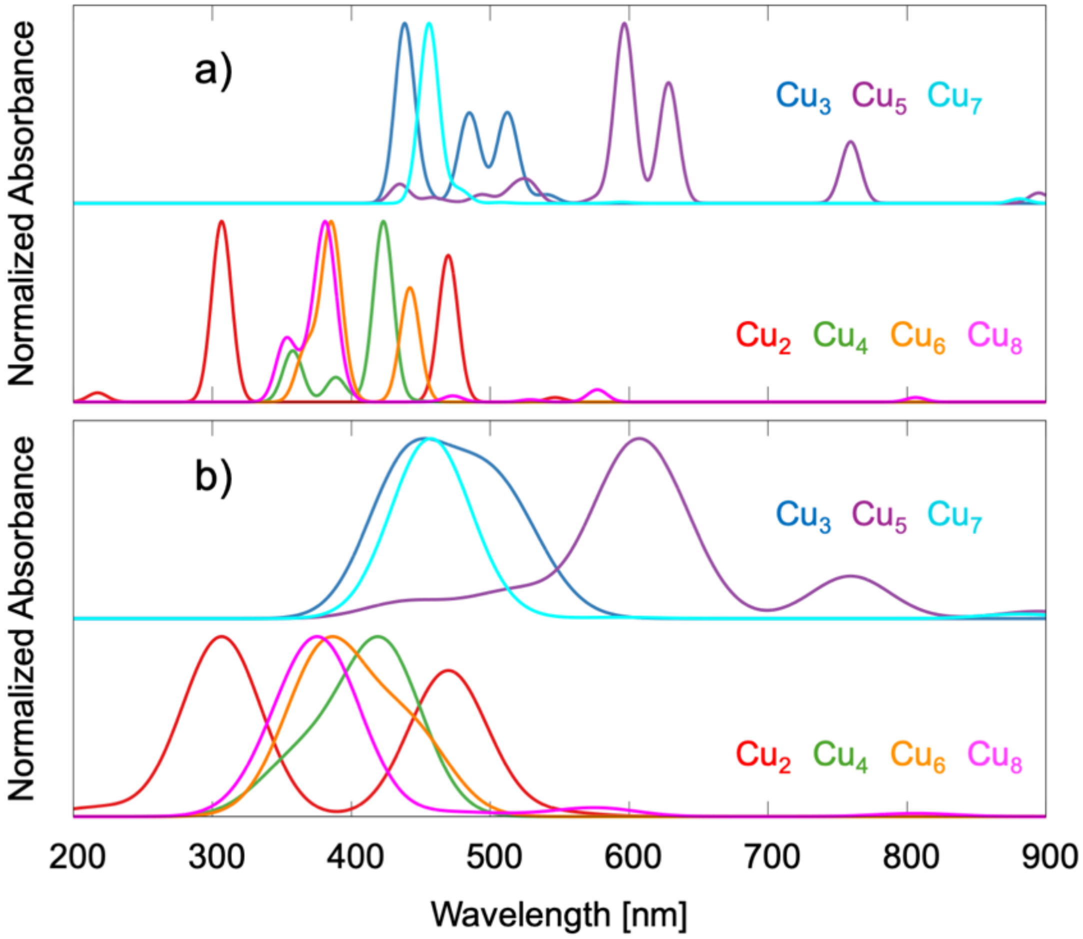

Figure S9. Calculated UV–Vis spectra of the modeled bare Cu_x system at the CAM-B3LYP/ Def2SVPP level of theory: (a) spectra with Gaussian broadening (10 nm half-width), and (b) spectra with a broader 40 nm half-width. This figure corresponds to **Figure 5** in the main manuscript but offers an alternative visualization.

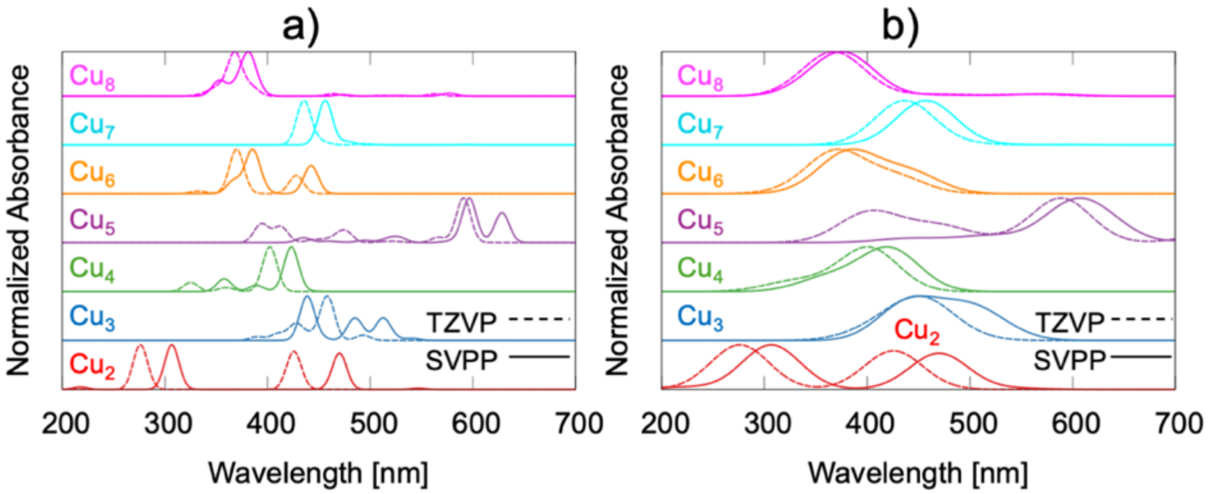

Figure S10. Calculated UV–Vis spectra of the modeled bare Cu_x system at the CAM-B3LYP level using two basis sets (Def2SVPP and Def2TZVP): (a) spectra with Gaussian broadening (10 nm half-width), and (b) spectra with a broader 40 nm half-width

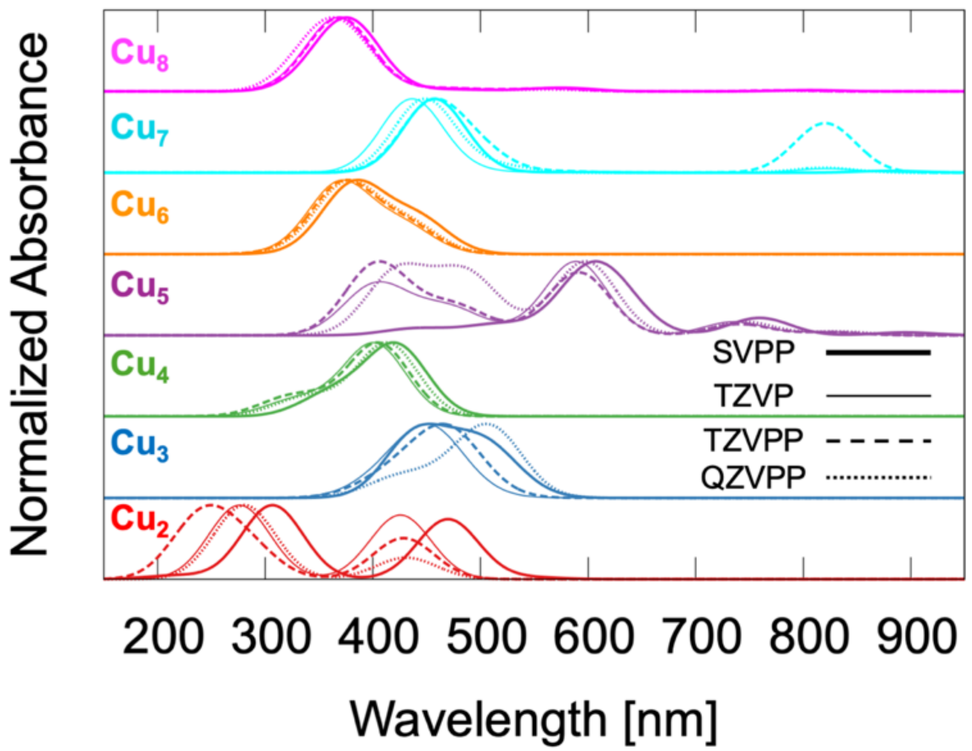

Figure S11. Calculated UV–Vis spectra of the modeled bare Cu_x system at the CAM-B3LYP level using four basis sets (Def2SVPP, Def2TZVP, Def2TZVPP, and Def2QZVPP). Spectra with a broader 40 nm half-width.

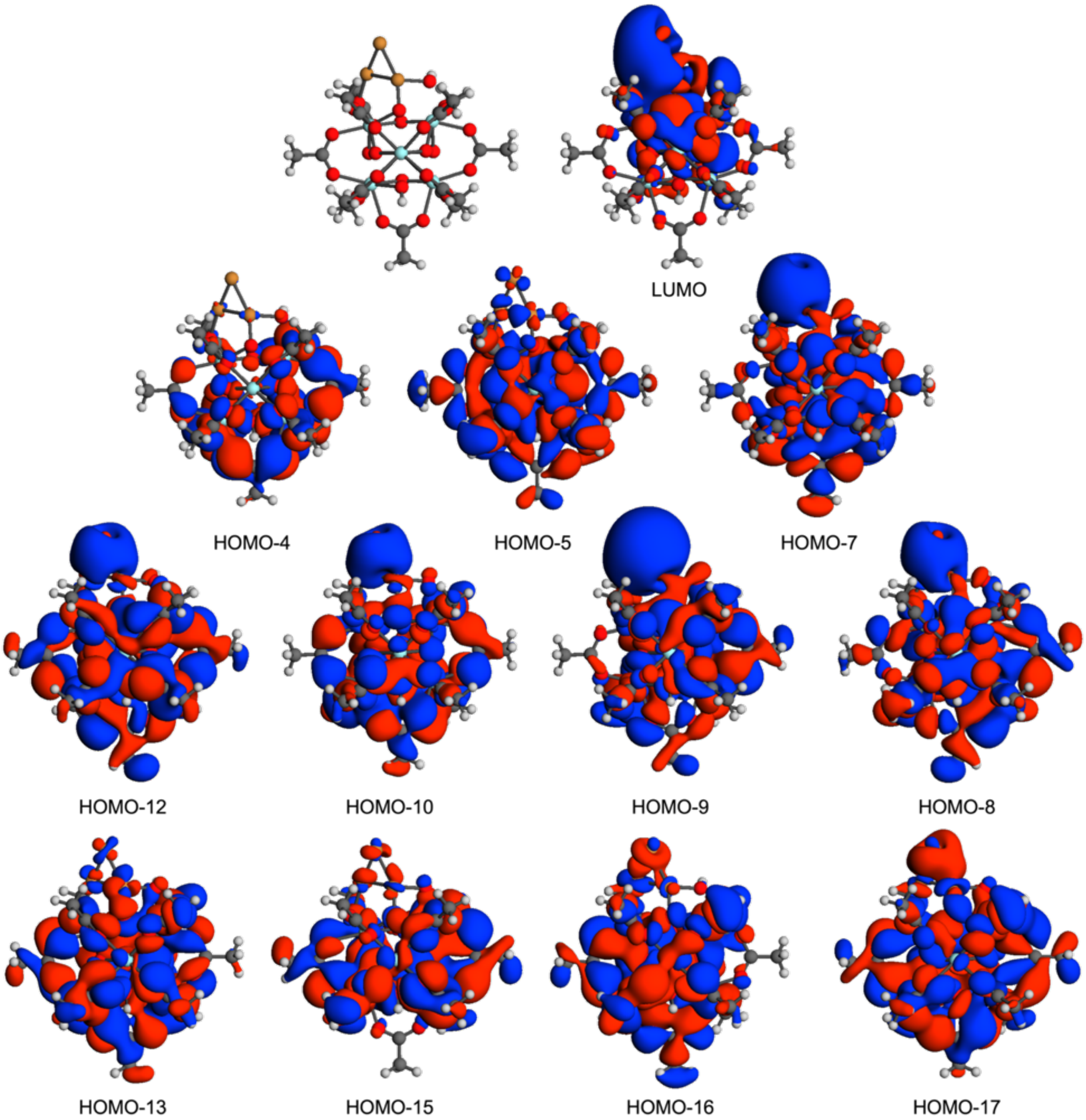

Figure S12. Geometry-optimized structure of the Cu_3-MOF model with the 3Cu(I) configuration featuring an acetate ligand, along with the dominant HOMOs and LUMO contributing to the corresponding computed UV–Vis given in **Fig. 6** in the main manuscript.

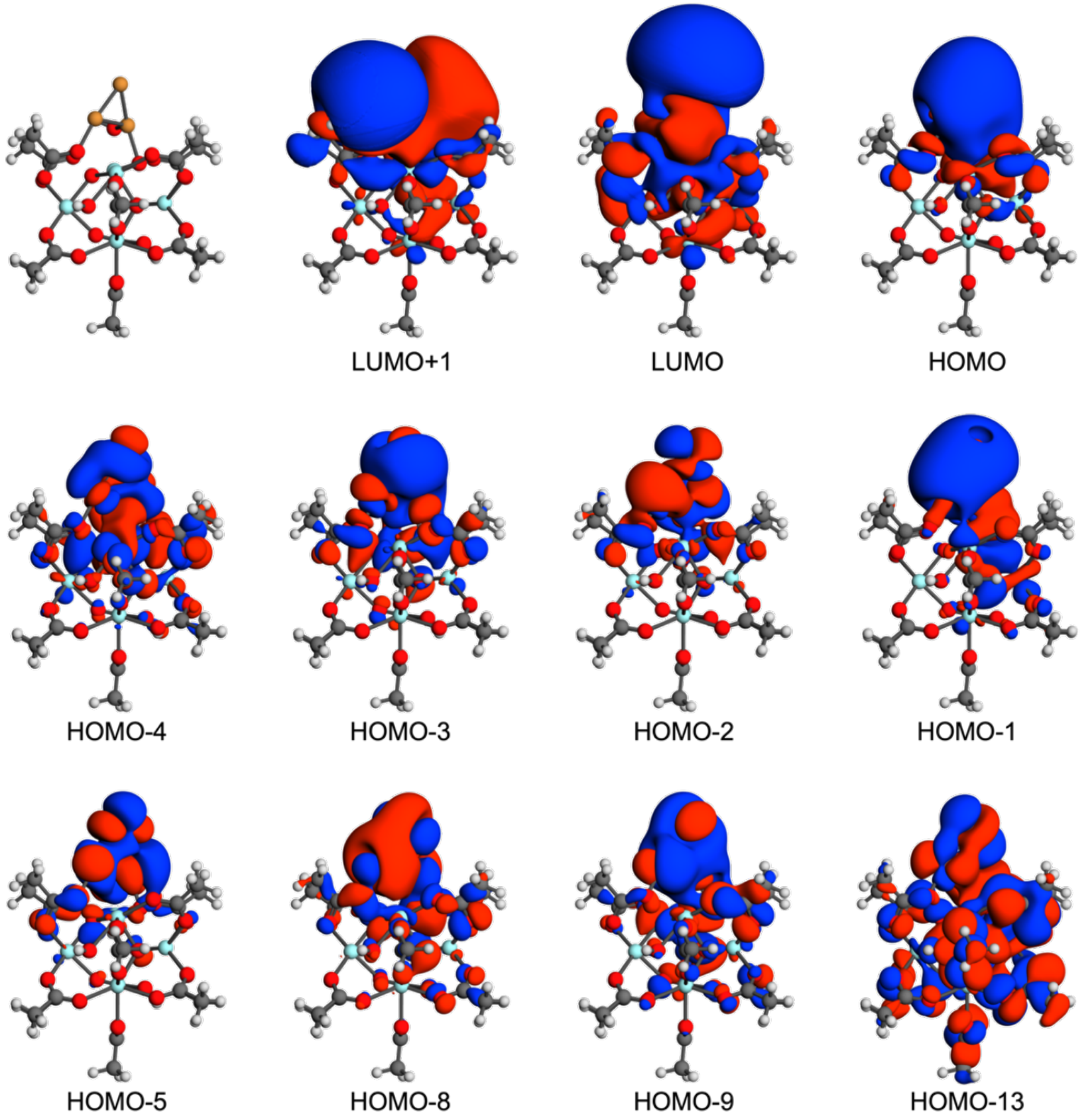

Figure S13. Geometry-optimized structure of the Cu_3-MOF model with the $2Cu^0$ + 1Cu(I) configuration featuring an acetate ligand, along with the dominant HOMOs and LUMOs contributing to the corresponding computed UV–Vis given in **Fig. 6** in the main manuscript.

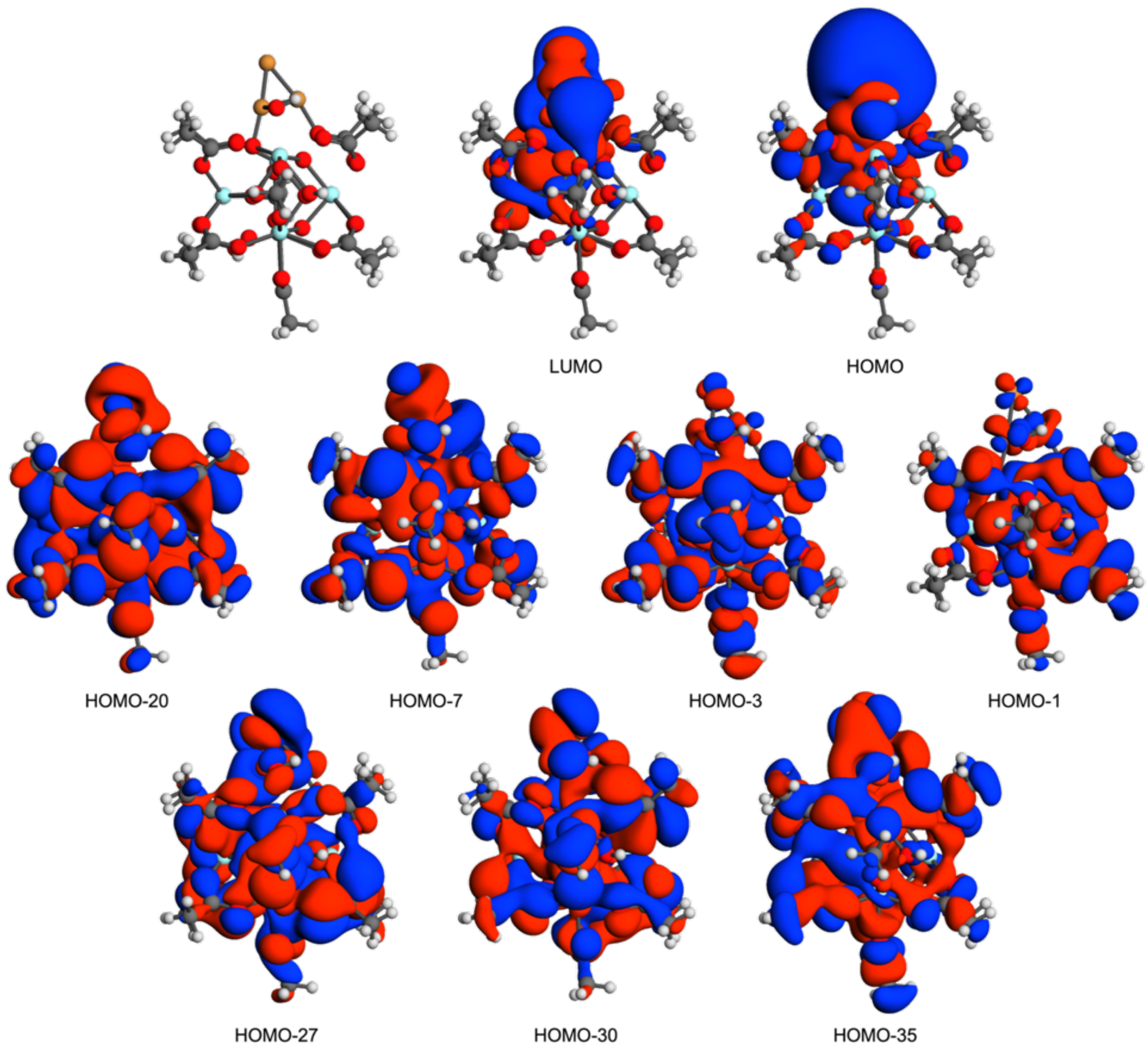

Figure S14. Geometry-optimized structure of the Cu_3-MOF model with the 2Cu(I) + $1Cu^0$ configuration featuring an acetate ligand, along with the dominant HOMOs and LUMOs contributing to the corresponding computed UV–Vis given in **Fig. 6** in the main manuscript.

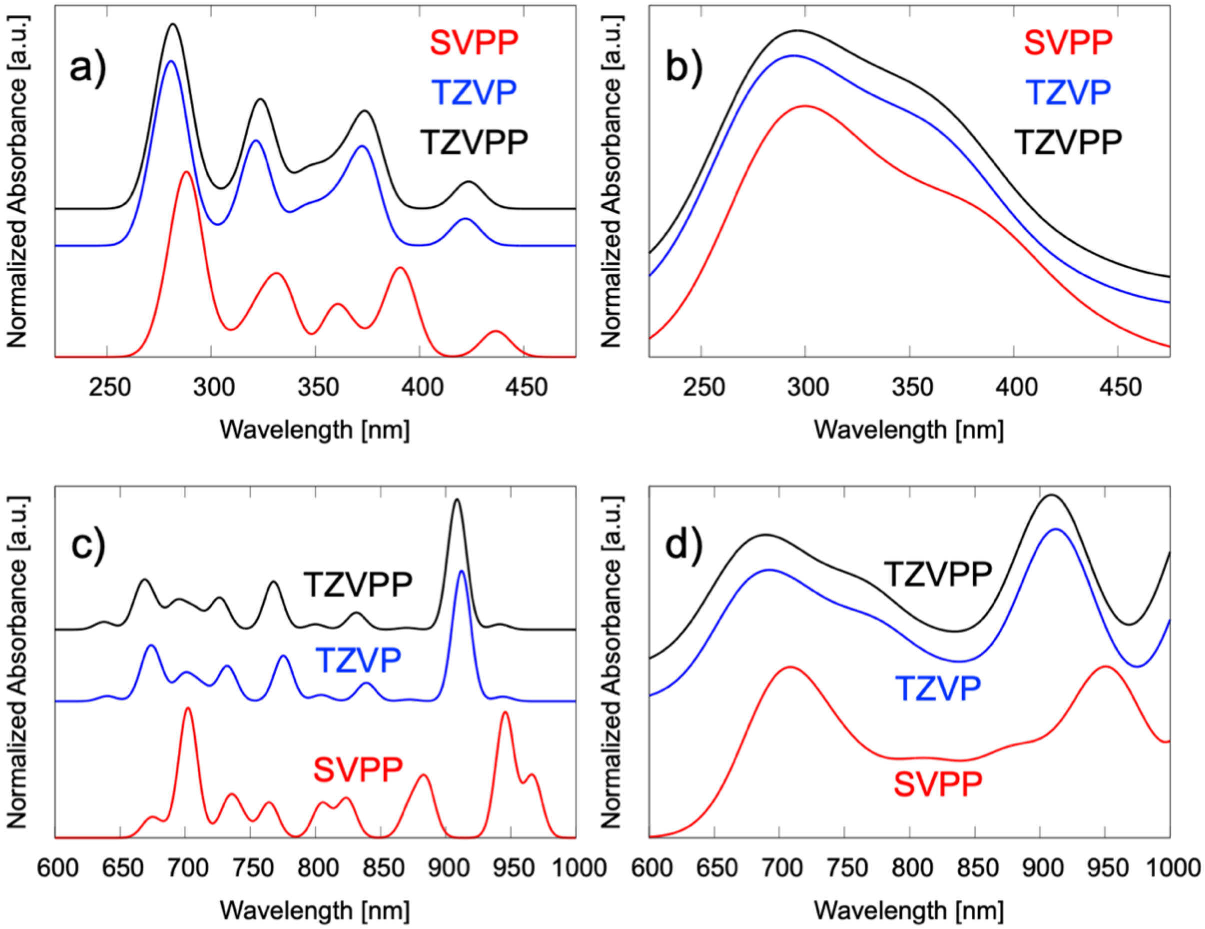

Figure S15. Calculated UV–Vis spectra of two selected configurations of the modeled Cu_3-MOF system with acetate linkers at the CAM-B3LYP level using different basis sets (Def2SVPP, Def2TZVP, and Def2TZVPP). Panels (a, b) correspond to the $2Cu^0$+1Cu(I) configuration, and panels (c,d) correspond to the 3Cu(I) configuration. Spectra in panels (a,c) are plotted with Gaussian broadening of10 nm half-width, while (b,d) use a broader 40 nm half-width.

2. Supporting Tables

Before examining the following tables, which summarize the **TD-DFT-calculated** absorption peaks and corresponding intensities for the **Cu_1–MOF, Cu_3–MOF,** and **bare Cu_x** cluster models (including representative examples of assigned electronic transitions), please note the following:

In situ UV-Vis Peaks for Cu_1/UiO-66 (before H_2 reduction)

- 230, 260, 284, 297 nm: $O^{2-} \rightarrow Cu^{2+}$ charge transfer (CT), consistent with earlier reports on similar catalysts.[1,2]
- 387 nm: oxo-bridged dimeric Cu sites.

In situ UV-Vis Peaks for Cu_x/UiO-66 (before H_2 reduction)

- 230, 260, 284, 297 nm: $O^{2-} \rightarrow Cu^{2+}$ charge transfer (CT), previously reported as 239, 267, 297 nm (assigned to Cu^{+}-like states) or 230, 263, 283, 298 nm (slightly blue-shifted).[3]

In situ UV-Vis Peaks for Cu_x/UiO-66 (after H_2 reduction)

- Same peaks at before H_2 reduction in addition to new band at ~567 nm, assigned to plasmon resonance of metallic Cu clusters.[4]

Refernces

(1) Impeng, S.; Salaya-Gerónimo, E.; Kunkel, B.; Bartling, S.; Faungnawakij, K.; Rungtaweevoranit, B.; Abdel-Mageed, A. M. Mechanism and Selectivity of MOF-Supported Cu Single-Atom Catalysts for Preferential CO Oxidation. J. Mater. Chem. A 2024, 12 (5), 3084–3095. https://doi.org/10.1039/D3TA05047E.

(2) Gurin, V. S.; Petranovskii, V. P.; Pestryakov, A. N.; Kryazhov, A.; Ozhereliev, O.; Hernandes, M.-A.; Alexeenko, A. A. Copper Clusters and Small Particles Stabilized within Nanoporous Materials. Eur. Phys. J. - At. Mol. Opt. Phys. 2003, 24 (1–3), 381–384. https://doi.org/10.1140/epjd/e2003-00195-4.

(3) Makhmutov, D.; Rungtaweevoranit, B.; Ahmed, A. A.; Faungnawakij, K.; Al-Yusufi, M.; Salaya, E.; Wohlrab, S.; Armbruster, U.; Abdel-Mageed, A. M. Catalytic and Kinetic Isotope Effect Studies of CO2 Reduction on Cu-Metalated UiO-66 Metal-Organic Framework. Appl. Surf. Sci. 2025, 688, 162323. https://doi.org/10.1016/j.apsusc.2025.162323.

(4) Pestryakov, A. N.; Petranovskii, V. P.; Kryazhov, A.; Ozhereliev, O.; Pfänder, N.; Knop-Gericke, A. Study of Copper Nanoparticles Formation on Supports of Different Nature by UV–Vis Diffuse Reflectance Spectroscopy. Chem. Phys. Lett. 2004, 385 (3–4), 173–176. https://doi.org/10.1016/j.cplett.2003.12.077.

UV-Vis Peaks: Cu_1–MOF Models

Table S1. Calculated absorption peaks [wavelength λ (nm) and intensity I (a.u.)] of the Cu_1–MOF model (Cu(I), acetate ligands) using the Def2SVPP basis set with various DFT functionals.

B3LYP		LC-ωHPBE		ωB97XD		CAM-B3LYP	
λ (nm)	I ×10^5	λ (nm)	I ×10^5	λ (nm)	I ×10^5	λ (nm)	I ×10^5
261	1.61	**210**	**8.22**	**220**	**6.37**	**221**	**6.75**
287	2.51	265	0.36	253	2.01	253	1.75
321	0.40	351	0.88	276	0.42	277	0.37
351	1.21	378	3.61	333	1.03	344	1.15
**388**	**4.78**	461	0.54	366	4.82	373	4.31
475	0.21			439	0.38	455	0.35

Table S2. Calculated absorption peaks [wavelength λ (nm) and intensity I (a.u.)] of the Cu_1–MOF model (Cu(I), acetate ligands) using the Def2TZVP basis set with various DFT functionals.

B3LYP		LC-ωHPBE		ωB97XD		CAM-B3LYP	
λ (nm)	I ×10^5	λ (nm)	I ×10^5	λ (nm)	I ×10^5	λ (nm)	I ×10^5
258	1.89	**205**	**8.45**	**216**	**7.80**	**215**	**8.08**
277	2.09	260	0.24	248	1.66	246	1.56
308	0.29	338	0.87	270	0.33	270	0.29
340	0.99	364	3.74	330	0.88	342	0.81
**379**	**4.74**	441	0.50	364	4.71	361	4.32
460	0.29			436	0.30	437	0.29

Notes

- All TD-DFT absorption peaks reported in the tables were obtained from spectra broadened with a 10 nm Gaussian, chosen to resolve most calculated transitions for meaningful comparison with experimental and literature data.
- For completeness, spectra were also computed with broader Gaussian widths (10–50 nm) to examine peak-shape evolution and enable comparison with the present in situ spectra. In both the main manuscript and the supporting information, figures are shown for spectra broadened by 10 and 40 nm.
- Bolded wavelengths and their corresponding intensities in the tables represent the most intense peaks for each individual spectrum.

Table S3. Calculated absorption peaks [wavelength λ (nm) and intensity I (a.u.)] for the Cu_1–MOF model (Cu(I), acetate ligands) using CAM-B3LYP with different basis sets. All cases use acetate ligands, except where noted for benzoate ligands.

SVPP		TZVP		TZVPP		SVPP (benzoate)	
λ (nm)	I ×10^5	λ (nm)	I ×10^5	λ (nm)	I ×10^5	λ (nm)	I ×10^5
**221**	**6.75**	**215**	**8.08**	**215**	**8.24**	**242**	**11.11**
253	1.75	246	1.56	246	1.56	287	0.47
277	0.37	270	0.29	271	0.28	344	1.12
344	1.15	332	0.81	334	0.79	379	3.63
373	4.31	361	4.32	364	4.25	465	0.30
455	0.35	437	0.29	440	0.27		

Table S4. Calculated absorption peaks [wavelength λ (nm) and intensity I (a.u.)] for the Cu_1–MOF model (Cu(II), acetate ligands) using the Def2SVPP basis set with various DFT functionals.

CAM-B3LYP		ωB97XD		LC-ω HPBE	
λ (nm)	I ×10^5	λ (nm)	I ×10^5	λ (nm)	I ×10^5
636	0.62	587	0.60	425	1.76
667	0.19	612	0.10	449	0.64
682	0.14	635	0.17	**479**	**2.74**
721	0.20	657	0.15	530	0.84
759	0.08	688	0.13	580	1.35
832	0.12	736	0.28	672	0.03
**882**	**1.11**	**811**	**0.74**	718	0.01
977	0.04	871	0.34	860	0.18
		897	0.08		
		969	0.08		

UV-Vis Peaks: Bare Cu_x Clusters

Table S5. Calculated absorption peaks [wavelength λ (nm) and intensity I (a.u.)] for the bare Cu_x clusters using CAM-B3LYP with different basis sets.

**Cu_x**	**SVPP**		**TZVP**		**TZVPP**		**QZVPP**	
	λ (nm)	I ×10^5	λ (nm)	I ×10^5	λ (nm)	I ×10^5	λ (nm)	I ×10^5
**Cu_2**	218	1.55	**276**	**31.64**	**241**	**38.17**	**280**	**75.27**
	**307**	**30.55**	426	27.03	283	18.77	431	23.82
	470	24.80			429	25.24		
	547	0.76						
**Cu_3**	**439**	**2.07**	391	1.28	392	1.31	417	3.94
	485	1.04	428	5.84	431	4.69	438	3.13
	513	1.05	**458**	**14.92**	**469**	**15.55**	491	10.58
	540	0.10	493	1.75	498	3.63	**516**	**16.65**
**Cu_4**	358	18.38	325	13.60	327	13.19	335	14.25
	389	8.85	360	5.88	360	5.13	361	4.37
	**423**	**64.72**	**402**	**67.85**	**406**	**66.27**	**414**	**64.82**
**Cu_5**	435	0.46	395	2.43	399	4.73	422	4.43
	459	0.14	412	2.11	451	0.42	452	0.60
	495	0.21	474	1.61	478	2.05	488	3.84
	525	0.59	521	0.18	524	0.21	528	0.32
	**597**	**4.27**	567	0.67	**593**	**5.49**	575	0.35
	629	2.86	**591**	**5.65**	741	1.05	**599**	**5.28**
	760	1.46	736	1.12	825	0.30	745	1.01
	895	0.25	824	0.30			834	0.29
**Cu_6**	**386**	**57.55**	333	4.34	335	4.59	338	5.12
	443	36.41	**370**	**75.93**	**373**	**72.57**	**377**	**70.99**
			428	31.21	430	31.45	434	30.78
**Cu_7**	**456**	**24.85**	**436**	**15.67**	**454**	**1.25**	**448**	**15.80**
	508	0.13	494	0.39	497	0.42	518	1.57
	594	0.18	575	0.03	574	0.03	819	1.08
	881	0.64	823	0.91	821	0.94		
**Cu_8**	354	25.97	**368**	**51.39**	**370**	**50.66**	345	39.60
	**381**	**72.62**	421	0.01	466	2.90	**373**	**53.67**
	473	2.46	464	2.95	512	0.73	401	8.00
	529	0.95	512	0.74	566	2.77	445	0.47
	577	4.94	564	2.87	778	0.99	471	2.98
	806	1.81	777	1.02			516	1.04
							570	2.49
							781	0.95

UV-Vis Peaks: Cu_3–MOF Models

Table S6. Calculated absorption peaks [wavelength λ (nm) and intensity I (a.u.)] of the Cu_3–MOF model (acetate ligands) for 5 configurations ((3Cu⁰ in M2 and M4, 3Cu(I), 2Cu⁰ + 1Cu(I), and 2Cu(I) + 1Cu⁰)) at the CAM-B3LYP/Def2SVPP level of theory.

$3Cu^0$ (M2)		$3Cu^0$ (M4)		3Cu(I)		$2Cu^0$+1Cu(I)		2Cu(I)+$1Cu^0$	
λ (nm)	I ×10^5	λ (nm)	I ×10^5	λ (nm)	I ×10^5	λ (nm)	I ×10^5	λ (nm)	I ×10^5
412	1.88	621	0.6	675	0.28	**288**	**18.1**	**364**	**3.29**
469	3.99	**651**	**3.57**	**702**	**1.74**	332	8.2	420	1.7
491	2.19	690	1.58	736	0.59	361	5.18	446	0.48
526	4.57	728	0.18	764	0.47	390	8.74	460	0.48
544	2.04	761	0.38	806	0.47	436	2.52	506	0.21
**630**	**14.95**	806	0.18	824	0.54			648	1.74
732	2.42	878	0.24	883	0.84			758	0.09
808	10.97	909	0.54	946	1.68			918	0.2
				966	0.85				

Table S7. Calculated absorption peaks [wavelength λ (nm) and intensity I (a.u.)] of the Cu_3–MOF model (benzoate ligands) for 4 configurations ((3Cu⁰ in M2 and M4, 3Cu(I), 2Cu⁰ + 1Cu(I), and 2Cu(I) + 1Cu⁰)) at the CAM-B3LYP/Def2SVPP level of theory.

$3Cu^0$ (M2)		$3Cu^0$ (M4)		3Cu(I)		2Cu(I)+$1Cu^0$	
λ (nm)	I ×10^5	λ (nm)	I ×10^5	λ (nm)	I ×10^5	λ (nm)	I ×10^5
543	1.08	**890**	**3.72**	804	0.95	388	0.62
**605**	**7.23**	952	3.51	857	0.67	432	0.54
632	0.52	978	1.09	893	0.37	458	0.47
658	0.7			940	1.11	494	0.12
681	0.83			**998**	**4.03**	**651**	**2.18**
724	2.06					739	0.07
762	1.2					916	0.22
796	1.46						
834	1.02						
972	2.1						

Table S8. Calculated absorption peaks [wavelength λ (nm) and intensity I (a.u.)] for the Cu_3–MOF model with acetate ligands in the $2Cu^0$ + 1Cu(I) configuration using CAM-B3LYP and different basis sets.

SVPP		TZVP		TZVPP	
λ (nm)	I ×10^5	λ (nm)	I ×10^5	λ (nm)	I ×10^5
**288**	**18.1**	**281**	**16.96**	**282**	**16.69**
332	8.2	322	9.66	324	9.93
361	5.18	372	9.16	374	8.87
390	8.74	422	2.5	424	2.46
436	2.52				

Table S9. Calculated absorption peaks [wavelength λ (nm) and intensity I (a.u.)] for the Cu_3–MOF model with acetate ligands in the 3Cu(I) configuration using CAM-B3LYP and different basis sets.

SVPP		TZVP		TZVPP	
λ (nm)	I ×10^5	λ (nm)	I ×10^5	λ (nm)	I ×10^5
675	0.28	640	0.12	638	0.17
**702**	**1.74**	674	1.27	669	1.12
736	0.59	701	0.66	696	0.68
764	0.47	732	0.8	726	0.72
806	0.47	776	1.03	768	1.07
824	0.54	804	0.14	800	0.12
883	0.84	839	0.42	832	0.38
946	1.68	872	0.04	870	0.04
966	0.85	**912**	**2.95**	**909**	**2.91**
		944	0.1	942	0.12

3. Supporting Notes

Note S1: UV–Vis Spectrum Assignment for the Cu_1–MOF Model: Cu(I)-Benzoate

Upon substituting the acetate ligand with a benzoate linker, the UV–Vis spectrum computed at the CAM-B3LYP/Def2SVPP level reveals a noticeable red-shift accompanied by a reduction in the number of well-resolved transitions. Specifically, five prominent absorption peaks are observed at 242, 287, 345, 380, and 465 nm, with the most intense band centered at 242 nm (see **Fig. 4c** in the main manuscript). This spectral evolution is attributed to the extended π-conjugation of the benzoate moiety, which facilitates greater electronic delocalization and enhances both ligand-to-metal charge transfer (LMCT) and intra-ligand π→π* excitations.

The intense 242 nm band arises primarily from transitions involving HOMO–16 → LUMO+7, LUMO+8, and LUMO+9; HOMO–14 → LUMO+21; HOMO–9 and HOMO–8 → LUMO+9; and HOMO–7 → LUMO+25, **see Figs. S6-S7** and **Table S10**. These transitions are predominantly characterized by π→π* excitations localized on the benzoate linkers, with only minor contributions from MLCT involving Cu d-orbitals. The absorption at 287 nm originates mainly from HOMO → LUMO+1 and HOMO → LUMO+14 transitions, corresponding to a combination of MLCT and d–d transitions.

The band at 345 nm is assigned to transitions from HOMO–26 → LUMO, LUMO+1, and LUMO+2, as well as from HOMO–17 → LUMO and LUMO+1, reflecting a mixed character of MLCT, LMCT, and d–d excitations (**Figs. S6-S7**). The 380 nm peak involves transitions from HOMO–2 → LUMO+1 and HOMO–1 → LUMO, LUMO+1, and LUMO+2, and is predominantly MLCT in nature. Finally, the broad feature at 465 nm is attributed to HOMO → LUMO, LUMO+1, LUMO+2, and LUMO+3 transitions, also corresponding mainly to MLCT and d–d contributions.

These results highlight the pronounced sensitivity of Cu(I) excited states to the electronic characteristics of their coordinating ligands, underscoring the pivotal influence of π-conjugation in tuning the optical response of MOF-supported copper catalysts. They also provide a compelling explanation for the wide variation in UV–Vis spectral signatures reported for Cu(I) species in the literature, much of which can be attributed to differences in ligand environments. This understanding offers a valuable framework for the future rational design of metalated MOFs, enabling targeted tailoring of their electronic properties through strategic ligand selection.

Table S10. Assignment of CAM-B3LYP/Def2SVPP-calculated absorption peaks for the Cu_1–MOF model with Cu(I) and both acetate and benzoate ligands.

**Acetate**	
λ (nm)	Assignment
**221**	**Cu-centered d–d, Cu → Zr MMCT, Cu → carboxylate MLCT**
253<br>277	Cu-centered d–d, MMCT, MLCT
344	Cu-centered d–d (dominant), minor node/linker contributions
373<br>455	Cu-centered d–d
**Benzoate**	
λ (nm)	Assignment
**242**	**π→π* excitations on benzoate linkers, minor MLCT from Cu d-orbitals**
287	MLCT, d–d transitions
345	MLCT, LMCT, d–d
380	predominantly MLCT
465	MLCT, d–d contributions

Note S2: Assignment of the UV–Vis Spectrum for the Cu_3–MOF Model (3Cu(I))

The UV–Vis spectrum of the fully oxidized 3Cu(I)-MOF model exhibits multiple low-energy absorption features spanning the near-infrared to visible region (675–966 nm), as shown in **Fig. 6a–b** in the main manuscript. These features arise exclusively from transitions into the LUMO, with two dominant peaks at 703 nm and 946 nm. The 703 nm band, primarily associated with the HOMO–4 → LUMO transition, corresponds mainly to LMCT from the carboxylate groups to the Cu_3 cluster and Zr ions, with minor π→π* contributions, see **Fig. S12** and **Table S11**. The 946 nm peak originates from combined HOMO–9, HOMO–5, and HOMO–4 → LUMO transitions, involving LMCT, π→π*, and d–d excitations within the Cu_3 cluster. Additional features at 883, 824, 806, 765, 736, and 675 nm arise from other occupied orbitals (e.g., HOMO–10, HOMO–12,

HOMO–13, HOMO–15, HOMO–17) transitioning to the LUMO, reflecting a rich mixture of MLCT, d–d, and intra-ligand π→π* transitions. This spectral complexity highlights the key role of multinuclear copper centers and linker electronic structure in modulating the optical properties of MOF-based systems.

Table S11. Assignment of CAM-B3LYP/Def2SVPP-calculated absorption peaks for the Cu_3–MOF model for both $3Cu^0$ M2 and 3Cu(I) configurations.

**$3Cu^0$ M2**	
λ (nm)	Assignment
476	Cu_3-centered MMCT, Cu→Zr MMCT
491	Cu_3-centered MMCT, minor Cu→Zr MMCT
526<br>546	Cu→Zr and Cu→carboxylate MLCT, coupled with MMCT
**630**	**Cu_3-centered MMCT, minor Cu→Zr MMCT**
**3Cu(I)**	
λ (nm)	Assignment
675	MLCT and Cu_3-centered MMCT
**703**	LMCT (carboxylate → Cu_3, Zr) with minor π→π* contributions
736	Mixed MLCT and Cu_3-centered MMCT
765	Cu_3-centered MMCT and MLCT
806	MLCT and π→π*
824	π→π* with minor MLCT
883	MLCT, Cu_3-centered MMCT, π→π*

Note S3: Assignment of the UV–Vis Spectra for Mixed-Valence Cu_3–MOF Models

The mixed-valence $2Cu^0$ + 1Cu(I) model exhibits a distinctive UV–Vis absorption profile, characterized by intense electronic transitions spanning the near-UV to visible region, with prominent peaks at 289, 332, 361, and 391 nm, followed by a sharp decline in intensity beyond 450 nm (**Fig. 6a–b** in the main manuscript). These features originate from partially delocalized Cu–Cu MMCT and Cu–ligand π* MLCT excitations.

Before discussing the individual transitions, it is important to note that the occupied molecular orbitals from HOMO to HOMO–9 are dominated by **Cu_3 d character** with **minor ligand and Zr** contributions, whereas HOMO–13 exhibits a **balanced Cu_3 d and ligand π composition**. Both LUMO and LUMO+1 are primarily **Cu_3-centered d orbitals, with minor Zr and ligand π* character** (**Fig. S13** and **Table S12**).

The intense absorption at 289 nm arises mainly from HOMO–13 and HOMO–8 → LUMO transitions, with additional contributions from HOMO–8, HOMO–9, HOMO–5, and HOMO–4 → LUMO+1. The 332 nm band involves HOMO–9, HOMO–8, and HOMO–1 → LUMO transitions, along with HOMO–2, HOMO–1, and HOMO → LUMO+1 excitations.

The peaks at 391, 361, and 437 nm originate predominantly from HOMO–3 to HOMO → LUMO transitions, corresponding to low-energy Cu_3 d–d and MLCT excitations. Collectively, these transitions reveal strong electronic communication within the trinuclear Cu_3 cluster and effective coupling with the linker, underscoring the critical role of mixed-valence Cu sites in defining the MOF's photophysical properties.

Compared to the mixed-valence $2Cu^0$ + 1Cu(I) model, the 2Cu(I) + $1Cu^0$ configuration exhibits a broader and less intense UV–Vis absorption profile, extending from 360 to 920 nm, with distinct bands at 364, 420, and 648 nm, and additional weaker features at 447, 460, 507, 758, and 918 nm (**Fig. 6a–b** in the main manuscript).

The 648 nm band originates from HOMO–7, HOMO–3, and HOMO → LUMO transitions, representing a combination of Cu_3-centered d–d excitations with additional contributions from LMCT, MLCT, and intra-ligand π→π* charge transfers (**Fig. S14** and **Table S12**).

The 420 nm band involves HOMO–35, HOMO–30, HOMO–27, and HOMO–1 → LUMO transitions, reflecting comparable contributions from MMCT within the Cu_3 cluster, LMCT, and ligand-centered π→π* excitations.

The most intense feature, at 364 nm, arises primarily from HOMO–35 and HOMO–20 → LUMO transitions, dominated by LMCT and π→π* character, with a minor MMCT contribution.

Overall, these transitions highlight significant electronic delocalization within the Cu_3 cluster and strong coupling to the framework linkers, underscoring the structural and electronic flexibility of mixed-valence sites in modulating the MOF's photophysical behavior.

Table S12. Assignment of CAM-B3LYP/Def2SVPP-calculated absorption peaks for the Cu_3–MOF model for both $2Cu^0 + 1Cu(I)$ and $2Cu(I) + 1Cu^0$ configurations.

**$2Cu^0 + 1Cu(I)$**	
λ (nm)	Assignment
**289**	**Dominant Cu_3-centered MMCT and ligand → Cu_3 LMCT<br>Minor Cu → Zr and Cu_3 → ligand π* MLCT**
332	Dominant Cu_3-centered MMCT<br>Minor MLCT (Cu_3 → ligand π*) and weak Cu → Zr CT.
361<br>391<br>437	Cu_3-centered MMCT + MLCT
**$2Cu(I) + 1Cu^0$**	
λ (nm)	Assignment
**364**	**Dominant LMCT and π→π* and Minor MMCT**
420	Cu_3-centered MMCT, LMCT, and ligand-centered π→π*
648	Cu_3-centered MMCT, with LMCT, MLCT, and ligand-centered π→π*

Note S4: UV–Vis Spectra of Cu_3–MOF Models with Benzoate Ligands

The impact of ligand identity on the optical response of Cu_3-MOF was investigated by substituting acetate with benzoate linkers in four selected configurations, as illustrated in **Fig. 6c-d** in the main manuscript. For the $3Cu^0$ M2 configuration, the benzoate-based model retains the overall spectral shape of the acetate analogue but exhibits broader and slightly red-shifted features. Notable transitions are observed at 497, 543, 605, 724, and 796 nm, with the strongest absorption centered at 605 nm, modestly blue-shifted relative to the 630 nm maximum in the acetate-linked version. Additionally, transitions in the 650–800 nm region gain intensity, suggesting that the extended π-system of the benzoate ligand promotes greater delocalization and stabilizes CT states.

This trend continues across the $3Cu^0$ M4, 3Cu(I), and 2Cu(I) + $1Cu^0$ configurations. The high-spin $3Cu^0$ M4 configuration shows deeper NIR absorption bands at 891, 953, and 978 nm, reflecting enhanced electronic coupling and increased stabilization of high-spin, delocalized excited states due to π-conjugation between the Cu_3 core and benzoate ligands. In the 3Cu(I) case, CT-enhanced transitions appear at 804, 857, 893, 941, and 999 nm, with the latter being most intense.

The 2Cu(I) + $1Cu^0$ configuration features sharper and more intense transitions across the visible and NIR regions, notably at 388, 432, 457, 494, and a dominant peak at 651 nm (**Fig. 6c-d** in the main manuscript). This further underscores the role of benzoate in enhancing oscillator strength and facilitating delocalized MMCT and MLCT transitions. Overall, substituting acetate with benzoate consistently induces red shifts, broadens absorption features, and enhances low-energy transitions, indicating stronger electronic coupling and more delocalized excitation behavior in the π-extended framework.

Note S5: Effect of Basis Sets and DFT Functionals on UV–Vis Spectra of Cu_3–MOF Models

The influence of basis set size on the optical response of Cu_3 clusters in the UiO-66 framework was evaluated for two representative configurations, mixed-valence $2Cu^0$ + 1Cu(I) and neutral 3Cu(I), using CAM-B3LYP with Def2SVPP, Def2TZVP, and Def2TZVPP basis sets (**Fig. S15**).

For the $2Cu^0$ + 1Cu(I) model, all three basis sets produced nearly identical spectral profiles, with principal transitions at 281–282, 323–324, 373–374, and 422–424 nm (**Fig. S15a–b**). Triple-ζ basis sets (Def2TZVP, Def2TZVPP) introduced only slight blue shifts relative to Def2SVPP, without altering the number, spacing, or relative intensities of the peaks. A similar pattern was observed for the 3Cu(I) configuration, which exhibits moderate-intensity transitions across 675–966 nm, attributed to symmetry distortion and charge redistribution within the Cu_3 core (**Fig. S15c–d**). Spectra computed with Def2TZVP and Def2TZVPP closely match those obtained using Def2SVPP, aside from slight blue shifts and minor intensity variations. The main transitions shift slightly to ~669–674, 701–726, 768–775, and 909–913 nm, with the most intense absorption consistently centered around 910 nm.

These minor spectral differences suggest that, although the larger basis sets theoretically improve the treatment of polarization and electron delocalization, their practical influence on spectral resolution and qualitative insight is minimal. Accordingly, Def2SVPP provides a computationally efficient yet chemically reliable level of theory for capturing CT and MMCT features in Cu_3-MOF systems, making it particularly suitable for high-throughput simulations and mechanistic studies.

Functional benchmarking revealed clear performance differences. For both $2Cu^0$ + 1Cu(I) and 3Cu(I) models, M06L and B3LYP produced red-shifted spectra with broader, less distinct features and reduced band separation, indicating limited accuracy in describing Cu–Cu and long-range LMCT excitations. In contrast, CAM-B3LYP yielded sharper, well-resolved spectra, underscoring the superiority of range-separated hybrid functionals for modeling the excited-state electronic structure of Cu_3–MOF catalysts.

Note S6: Fit quality metrics: in situ spectra vs weighted TD-DFT-constructed spectra

The fit quality metrics comparing the experimental in situ UV–Vis spectra of Cu_1/UiO-66 and Cu_x/UiO-66 with their reconstructed counterparts, generated as weighted sums of TD–DFT-simulated spectra, are summarized in **Tables S13–S14**. For each spectrum, we report the Pearson correlation coefficient (r) with its 95 % confidence interval ($r_{95\%CI}$), together with the root-mean-square error (RMSE, in absorbance units), normalized RMSE (nRMSE), coefficient of determination R^2 and its adjusted form R^2_{adj}, spectral angle mapper (SAM), first-derivative correlation (r′), and optimal lag correlation (r_{opt}).

Pearson's r captures overall shape similarity, penalizing both peak position mismatches (x-axis) and intensity differences (y-axis). The nRMSE measures quantifies the residual magnitude relative to the observed range, while R^2_{adj} reflects the proportion of variance explained, adjusted for model complexity. SAM quantifies the angular similarity between spectra viewed as vectors, and r′emphasizes peak position alignment independent of baseline intensity. The r_{opt} metric represents the maximum correlation obtainable when allowing a small wavelength shift ($\Delta\lambda$, ±3 nm) between the reconstructed and experimental spectra, with $\Delta\lambda$ indicating the magnitude and direction of the shift (negative values mean the reconstructed spectrum is shifted toward shorter wavelengths).

For most spectra, $|\Delta\lambda| \leq 3$ nm, and the improvement from r to r_{opt} is minimal ($\Delta r \leq 0.01$), confirming that excellent agreement is achieved without significant spectral shifting. In a few Cu_x/UiO-66 cases, Δr is modestly higher ($\approx$ 0.03–0.04 at $\Delta\lambda \approx -2$ nm), suggesting a minor systematic offset consistent with experimental calibration tolerances and the well-known excitation-energy bias of TD–DFT.

For Cu_1/UiO-66, fit quality improves markedly with Gaussian broadening from 10 nm to 30–40 nm, see **Tables S13**. Narrow broadening (10 nm) yields the lowest r values (0.74–0.82) and highest nRMSE (> 0.15), reflecting limited agreement due to unresolved experimental peak widths. At 30–40 nm broadening, r exceeds 0.94 for all spectra, nRMSE falls below 0.08, and R^2_{adj} approaches 0.94, indicating excellent agreement in both shape and intensity. The best-performing case is EXP3 at 40 nm, with r = 0.973 (95% CI: 0.971–0.974), nRMSE = 0.062, R^2_{adj} = 0.941, the smallest SAM (0.146 rad), and r_{opt} = 0.974, and $\Delta\lambda$ = +3 nm (minimal gain from r to r_{opt} (Δr = 0.001) and small wavelength shift), confirming highly accurate peak position and intensity matching. These results

highlight the value of moderate spectral broadening to reconcile high-resolution TD-DFT features with experimental linewidths, improving both deconvolution and transition assignments.

For Cu_x/UiO-66, a similar trend is observed: Gaussian broadening from 10 nm to 40 nm steadily increases r and R^2_{adj} while reducing nRMSE, see **Tables S14**. At 10 nm, r remains in the 0.68–0.71 range with nRMSE between 0.08 and 0.14, indicating that narrow broadening fails to reproduce the experimental envelopes of overlapping transitions. Broadening to 30–40 nm reduces residuals significantly, with r surpassing 0.90 in most cases and nRMSE dropping below 0.06 for the best fits. The highest-quality match is obtained for EXP4 at 40 nm (r = 0.952, 95% CI: 0.950–0.954; nRMSE = 0.057; R^2_{adj} = 0.889; SAM = 0.172 rad), reflecting close agreement in spectral shape and relative intensities.

EXP1 = before H_2 reduction at 30 °C, EXP2 = before H_2 reduction at 250 °C, EXP3 = during 2 h of H_2 reduction, and EXP4 = after H_2 reduction at 30 °C.

Table S13. Fit quality metrics for comparing reconstructed spectra (obtained as weighted sums of TD-DFT-simulated spectra) with the corresponding experimental in situ UV–Vis spectra of the Cu_1/UiO-66 catalyst under the specified conditions (EXP1–EXP4).

Experiment & Broadening (nm)		r	$r_{95\%CI}$	R^2	R^2_{adj}	RMSE	nRMSE	r_{opt}	Δλ	SAM	r′
EXP1	10	0.821	0.812–0.829	0.663	0.828	0.332	0.154	0.828	-3	0.484	-0.028
	20	0.933	0.929–0.936	0.869	0.935	0.207	0.096	0.935	-3	0.294	0.106
	30	0.952	0.949–0.954	0.900	0.953	0.181	0.084	0.953	3	0.257	0.126
	40	0.944	0.941–0.947	0.883	0.946	0.195	0.090	0.946	3	0.277	0.119
EXP2	10	0.820	0.812–0.828	0.662	0.661	0.406	0.154	0.825	-3	0.478	-0.002
	20	0.935	0.931–0.938	0.873	0.873	0.249	0.094	0.939	-3	0.286	0.116
	30	0.963	0.961–0.965	0.921	0.921	0.197	0.074	0.964	-3	0.225	0.142
	40	0.960	0.958–0.962	0.912	0.911	0.208	0.079	0.961	3	0.238	0.139
EXP3	10	0.756	0.744–0.766	0.494	0.492	0.428	0.181	0.762	-3	0.443	-0.015
	20	0.915	0.911–0.919	0.833	0.832	0.246	0.104	0.922	-3	0.249	0.082
	30	0.968	0.966–0.969	0.936	0.935	0.153	0.065	0.970	-3	0.154	0.106
	40	0.973	0.971–0.974	0.945	0.941	0.145	0.062	0.974	3	0.146	0.112
EXP4	10	0.745	0.733–0.756	0.462	0.460	0.410	0.187	0.753	-3	0.439	-0.017
	20	0.914	0.910–0.918	0.829	0.829	0.231	0.106	0.921	-3	0.241	0.094
	30	0.967	0.966–0.969	0.935	0.935	0.142	0.065	0.969	-3	0.148	0.120
	40	0.972	0.971–0.974	0.940	0.940	0.137	0.063	0.974	3	0.142	0.128

Table S14. Fit quality metrics for comparing reconstructed spectra (obtained as weighted sums of TD-DFT-simulated spectra) with the corresponding experimental in situ UV–Vis spectra of the Cu_x/UiO-66 catalyst under the specified conditions (EXP1–EXP4).

Experiment & Broadening (nm)		r	$r_{95\%CI}$	R^2	R^2_{adj}	RMSE	nRMSE	r_{opt}	Δλ	SAM	r′
EXP1	10	0.709	0.696–0.722	0.498	0.497	0.341	0.088	0.722	-3	0.574	-0.011
	20	0.816	0.807–0.824	0.655	0.654	0.283	0.073	0.826	-3	0.467	-0.001
	30	0.837	0.829–0.844	0.673	0.672	0.276	0.071	0.844	-3	0.454	0.002
	40	0.826	0.818–0.834	0.654	0.653	0.284	0.073	0.835	-3	0.468	0.005
EXP2	10	0.680	0.666–0.693	0.400	0.398	0.403	0.048	0.722	-3	0.518	-0.064
	20	0.816	0.808–0.825	0.664	0.663	0.302	0.036	0.826	-3	0.379	-0.088
	30	0.871	0.865–0.877	0.755	0.754	0.257	0.031	0.844	-3	0.322	-0.098
	40	0.873	0.867–0.879	0.754	0.753	0.258	0.031	0.835	-3	0.323	-0.109
EXP3	10	0.697	0.683–0.709	0.399	0.397	0.373	0.079	0.707	3	0.470	0.003
	20	0.852	0.845–0.859	0.724	0.723	0.253	0.054	0.856	3	0.312	0.013
	30	0.908	0.903–0.912	0.816	0.815	0.206	0.044	0.910	3	0.253	0.017
	40	0.910	0.906–0.915	0.810	0.809	0.210	0.045	0.911	3	0.258	0.020
EXP4	10	0.682	0.668–0.695	0.316	0.314	0.358	0.143	0.707	3	0.441	-0.019
	20	0.876	0.870–0.882	0.759	0.758	0.213	0.085	0.856	3	0.256	-0.004
	30	0.943	0.940–0.946	0.885	0.885	0.147	0.059	0.910	3	0.176	-0.001
	40	0.952	0.950–0.954	0.890	0.889	0.144	0.057	0.911	3	0.172	0.001

Note S7: Universality and Transferability of the DR–UV–Vis + TD–DFT Approach

Although this study focuses on Cu, the DR–UV–Vis + TD–DFT fingerprinting workflow is inherently general and transferable to other transition metals in MOFs (e.g., Fe, Co, Ni, Mn), provided that diagnostically useful transitions fall within the experimental spectral window and are sufficiently intense in diffuse reflectance. Accurately capturing these features requires considering all chemically relevant oxidation states and spin configurations (e.g., low- vs. high-spin Fe^{2+}, Co^{2+}), as these strongly influence geometry and spectral signatures.

For open-shell centers, spin-polarized TD–DFT is essential, and hybrid functionals such as CAM-B3LYP or PBE0 are generally preferred for their reliability in describing both d–d and CT excitations. Triple-ζ basis sets for the metal center (Def2TZVP or Def2TZVPP) combined with at least double-ζ for light atoms strike a practical balance between accuracy and cost. For certain ions, such as high-spin Mn(II) or Fe(III), d–d transitions are spin-forbidden and intrinsically weak, making CT features the primary diagnostics. In such cases, strong multiplet effects or electron correlation may necessitate complementary methods (e.g., CASSCF, ligand field theory) for reliable assignment.

In practice, the approach adapts by generating reference spectra for plausible site structures spanning oxidation states, spin multiplicities, and coordination environments, and by quantitatively matching these to experimental DR–UV–Vis data using fit-quality metrics such as the Pearson correlation coefficient (r) and those outlined in **Note S6**. This flexibility enables reliable fingerprinting of a wide range of metalated MOFs while explicitly accounting for the distinct electronic-structure characteristics of each metal.

Finally, the approach is grounded in the simulation of spectra acquired from real samples subjected to activation treatments relevant to their catalytic applications. Extending this methodology to other systems will therefore require corresponding experimental datasets, which form an integral part of our planned future work.
